# Supplementary material for: Postmortem memory of public figures in news and social media
Source: Proc Natl Acad Sci U S A. 2021 Sep 15;118(38):e2106152118. doi: 10.1073/pnas.2106152118 (PMC8463883; doi:10.1073/pnas.2106152118)
Supplement: Supplementary File [file pnas.2106152118.sapp.pdf]

Post-mortem memory of public figures in news and social media

Robert West, Jure Leskovec, Christopher Potts

Number of documents per day

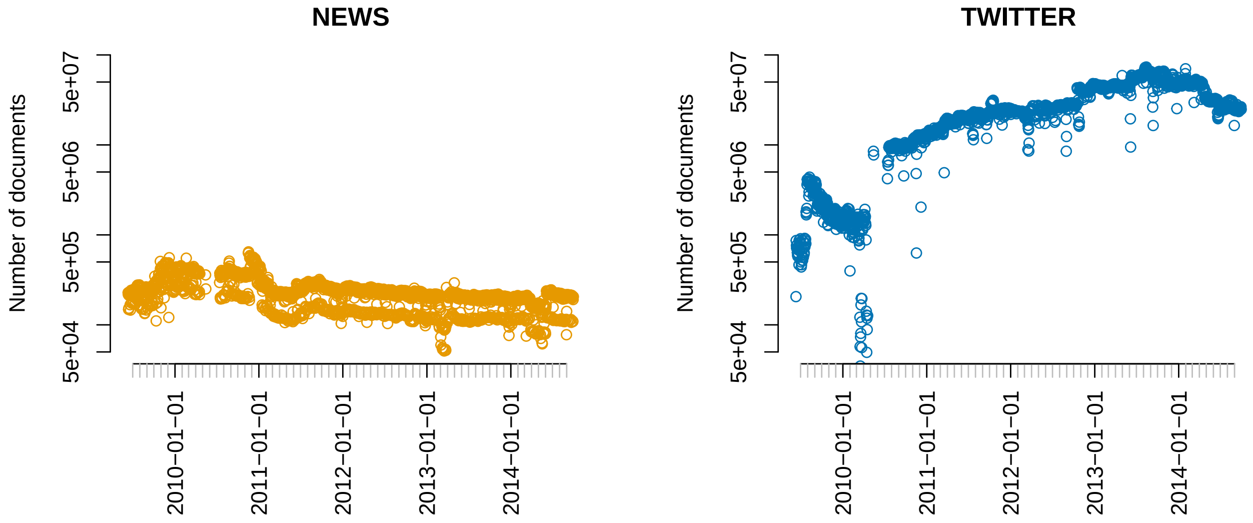

Supplementary Figure 1: Number of documents per day in the Spinn3r corpus.

Taxonomy of causes of death

Natural causes of death

Acute myeloid leukemia, Adrenocortical carcinoma, Alveolar rhabdomyosarcoma, Alzheimer's disease, Amyloidosis, Amyotrophic lateral sclerosis, Anemia, Aneurysm, Aortic aneurysm, Aortic dissection, Appendix cancer, Asthma, Astrocytoma, Atherosclerosis, Atypical teratoid rhabdoid tumor, B-cell chronic lymphocytic leukemia, Bladder cancer, Bleeding, Blood disorder, Blunt trauma, Bone cancer, Bone tumor, Brain Cancer, Brain damage, Brain tumor, Breast cancer, Bronchitis, Bronchopneumonia, Cancer, Cardiac arrest, Cardiac dysrhythmia, Cardiac surgery, Cardiopulmonary Arrest, Cardiovascular disease, Cerebral hemorrhage, Cerebral infarction, Cervical cancer, Cholangiocarcinoma, Chronic kidney disease, Chronic Obstructive Pulmonary Disease, Cirrhosis, Colorectal cancer, Complication, Complications from a stroke, Complications from cardiac surgery, Complications from pneumonia, Complications of diabetes mellitus, Congenital heart defect, Coronary artery disease, Craniocerebral Trauma, Creutzfeldt–Jakob disease, Cystic fibrosis, Dementia, Dementia with Lewy bodies, Diabetes mellitus, Disease, Ebola virus disease, Emphysema, Epileptic seizure, Esophageal cancer, Gallbladder cancer, Glioblastoma multiforme, Heart Ailment, Heart failure, Heart valve disease, Heat Stroke, Hepatitis, HIV/AIDS, Hodgkin's lymphoma, Huntington's disease, Hypertension, Hypertensive heart disease, Hyperthermia, Illness, Infection, Influenza, Internal bleeding, Intracranial aneurysm, Intracranial hemorrhage, Kidney cancer, Laryngeal cancer, Leiomyosarcoma, Leukemia, Liver cancer, Liver disease, Liver failure, Liver tumour, Lung cancer, Lung disease, Lung Infection, Lymphoma, Malaria, Melanoma, Meningitis, Mesothelioma, Metastatic breast cancer, Metastatic Melanoma, Motor neuron disease, Multiple myeloma,

Multiple organ dysfunction syndrome, Multiple organ failure, Multiple sclerosis, Multiple system atrophy, Myelodysplastic syndrome, Myocardial infarction, Natural causes, Nephropathy, Non-Hodgkin lymphoma, Old age, Oral cancer, Organ dysfunction, Ovarian cancer, Pancreatic cancer, Pancreatitis, Parkinson's disease, Peritonitis, Pneumonia, Pneumothorax, Polycythemia, Polymyalgia rheumatica, Progressive supranuclear palsy, prolonged illness, Prostate cancer, Pulmonary edema, Pulmonary embolism, Pulmonary failure, Pulmonary fibrosis, Pyelonephritis, Renal failure, Respiratory arrest, Respiratory disease, Respiratory failure, Salivary gland neoplasm, Sepsis, Septic shock, Skin cancer, Smallpox, Squamous-cell carcinoma, Stomach cancer, Stroke, Subarachnoid hemorrhage, Subdural hematoma, Surgery, Surgical complications, T-Cell Lymphoma, Terminal illness, Throat Cancer, Thrombosis, Thrombus, Thyroid cancer, Urinary tract infection, Uterine cancer, Vascular dementia, Viral pneumonia

Unnatural causes of death

Accident, Accidental drug overdose, Accidental fall, Airstrike, Alcohol intoxication, Ambush, Asphyxia, Assassination, Assisted suicide, Aviation accident or incident, Ballistic trauma, Bike accident, Blast injury, Boating Accident, Bomb, Brain injury, Capital punishment, Car bomb, Carbon monoxide poisoning, Casualty of war, Cocaine overdose, Combined drug intoxication, Decapitation, Drowning, Drug overdose, Execution, Execution by firing squad, Execution-style murder, Explosion, Falling, Falling from height, Fire, Firearm, Gunshot, Hanging, Helicopter crash, Heroin overdose, Hit and run, Homicide, Improvised bombing, Injury, Killed in action, Lethal injection, Lightning, Major trauma, Motorcycle accident, Mountaineering, Murder, Murder_suicide, Murder–suicide, Poison, Poisoning, Racing Accident, Self-inflicted wound, Shark attack, Shootout, Skiing accident, Smoke inhalation, Stab wound, Stabbing, Strangling, Struck by car, Suicide, Suicide attack, Suicide by hanging, Tornado Incident, Torture, Traffic collision, Traumatic brain injury

Taxonomy of notability types

- **academia/engineering:** Academic, Aircraft designer, Amusement Ride Designer, Astronaut, Astronomer, Computer Scientist, Honorary Degree Recipient, Invention, Inventor, Physician, Surgeon, Translator
- **art:** Architect, Author, Automotive Designer, Bassist, Blogger, Book, Broadcast Artist, Chef, Collector, Comic Book Colorist, Comic Book Creator, Comic Book Inker, Comic Book Letterer, Comic Book Penciler, Comic Book Writer, Comic Strip Creator, Composer, Conductor, Drummer, Fashion designer, Fictional Character Creator, Film actor, Film art director, Film casting director, Film cinematographer, Film costumer designer, Film crewmember, Film critic, Film director, Film editor, Film music contributor, Film producer, Film production designer, Film set decorator, Film story contributor, Film subject, Film writer, Game designer, Guitarist, Hobbyist, Illustrator, Lyricist, Museum director, Music video director, Music video performer, Musical Artist, Musician, Newspaper Owner, Opera Director, Opera singer, Periodical editor, Person or entity appearing in film, Record Producer, Recording Engineer, Ship Designer, Songwriter, Theater Actor, Theater Choreographer, Theater Designer, Theater Director, Theater Producer, TV Actor, TV Character, TV Director, TV Personality, TV Producer, TV Program Creator, TV program guest, TV station owner, TV subject, TV Writer, Video Game Actor, Video Game Designer, Visual Artist
- **general fame:** Appointee, Award Nominee, Award Winner, Celebrity, Competitor, Deity, Department, Event, Family member, Family name, Hall of fame inductee, Interviewee, Literature Subject, Noble person, Organization member, Person, Person Or Being In Fiction, Project participant, Quotation Subject, Shareholder, Social network user, Sponsored Recipient
- **known for death:** Deceased Person, Disaster survivor, Disaster victim
- **leadership:** Chivalric Order Member, Judge, Military Commander, Military Person, Monarch, Organization founder, Organization leader, Politician, Religious Leader, Religious Leadership Role, U.S. Congressperson
- **sports:** American football head coach, American football player, Athlete, Australian Rules Footballer, Baseball Coach, Baseball Manager, Baseball Player, Basketball Coach, Basketball Player, Boxer, Chess

Player, Cricket Bowler, Cricket Player, Cricket Umpire, Cyclist, Drafted athlete, Football player, Football team manager, Golf Course Architect, Golfer, Ice hockey coach, Ice hockey player, Martial Artist, Mountaineer, Olympic athlete, Sports League Award Winner, Sports official, Sports team coach, Sports Team Owner, Tennis Player, Tennis Tournament Champion

Biographic statistics of public figures

	**All**	**Included**	**Regression**
**Age**			
N/A	10%	6%	0%
1st quartile	68	64	60
Mean	76	74	70
Median	80	77	73
3rd quartile	88	87	84
**Gender**			
N/A	27%	7%	0%
Female	16%	17%	17%
Male	84%	83%	83%
**Manner of death**			
N/A	76%	60%	0%
Natural	85%	86%	88%
Unnatural	15%	14%	12%
**Language**			
N/A	45%	27%	14%
Anglophone	60%	82%	80%
Non-anglophone	40%	18%	20%
**Notability type**			
N/A	1%	0%	0%
Arts	40%	50%	56%
Sports	14%	14%	14%
Leadership	11%	14%	13%
Known for death	26%	16%	10%
General fame	7%	4%	5%
Academia/engineering	2%	2%	2%
**Count**	33 340	2 362	870

Supplementary Table 1: Biographic statistics of public figures. *All* refers to the 33,340 individuals listed in the Freebase knowledge base as having died between 11 June 2009 and 30 September 2014. *Included* refers to the 2,362 individuals included in the study (exclusion was mostly due to below-threshold pre-mortem mention frequencies, cf. *Materials and Methods* in the main paper). *Regression* is a subset of *Included* and refers to the 870 individuals included in the regression analysis (exclusion was due to unknown age, gender, or manner of death). *N/A* refers to the percentage of individuals for whom the respective property was not available in Freebase. The remaining relative frequencies were computed based on the set of individuals for whom the property was available, so they sum to 100%.

Model comparison

(a) News

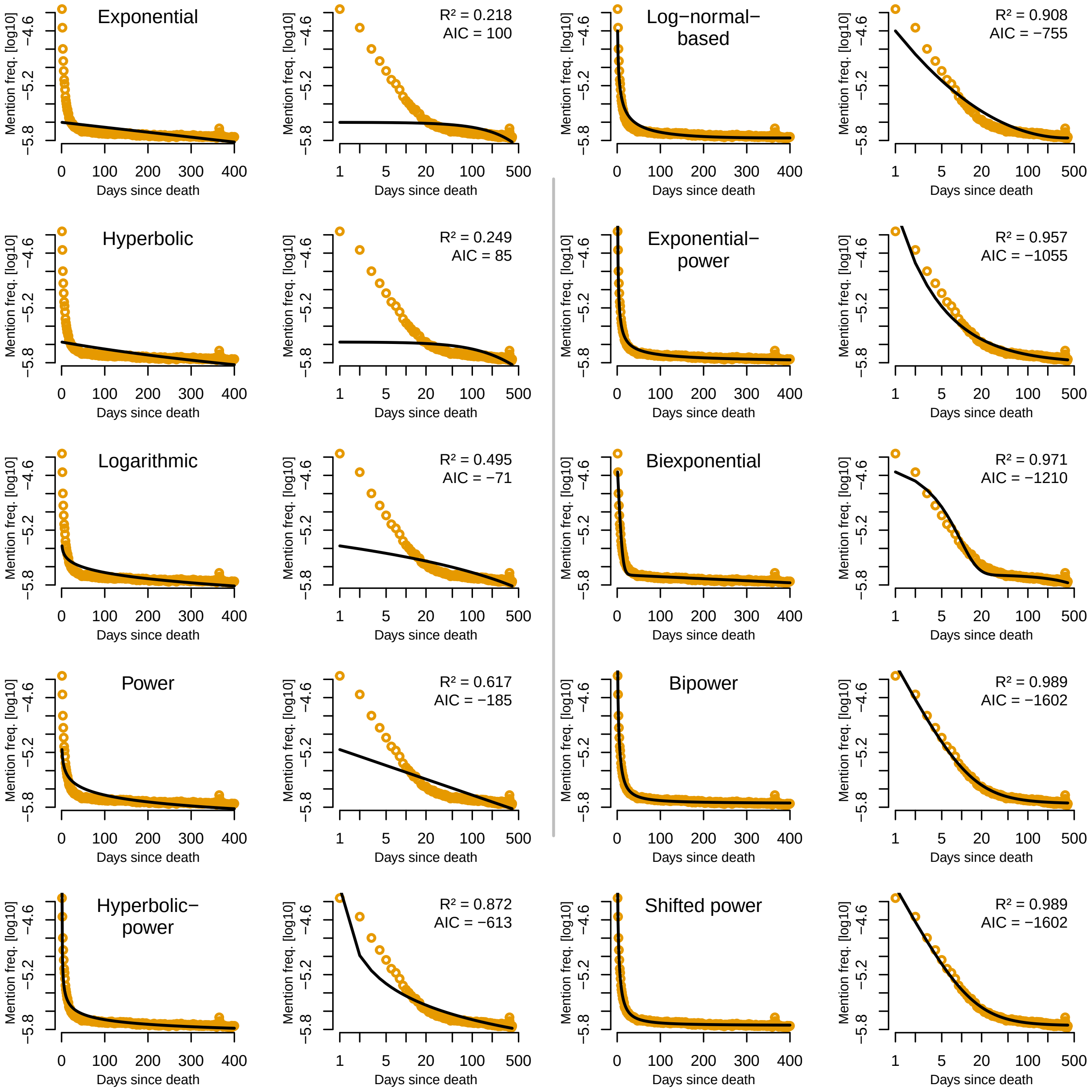

(b) Twitter

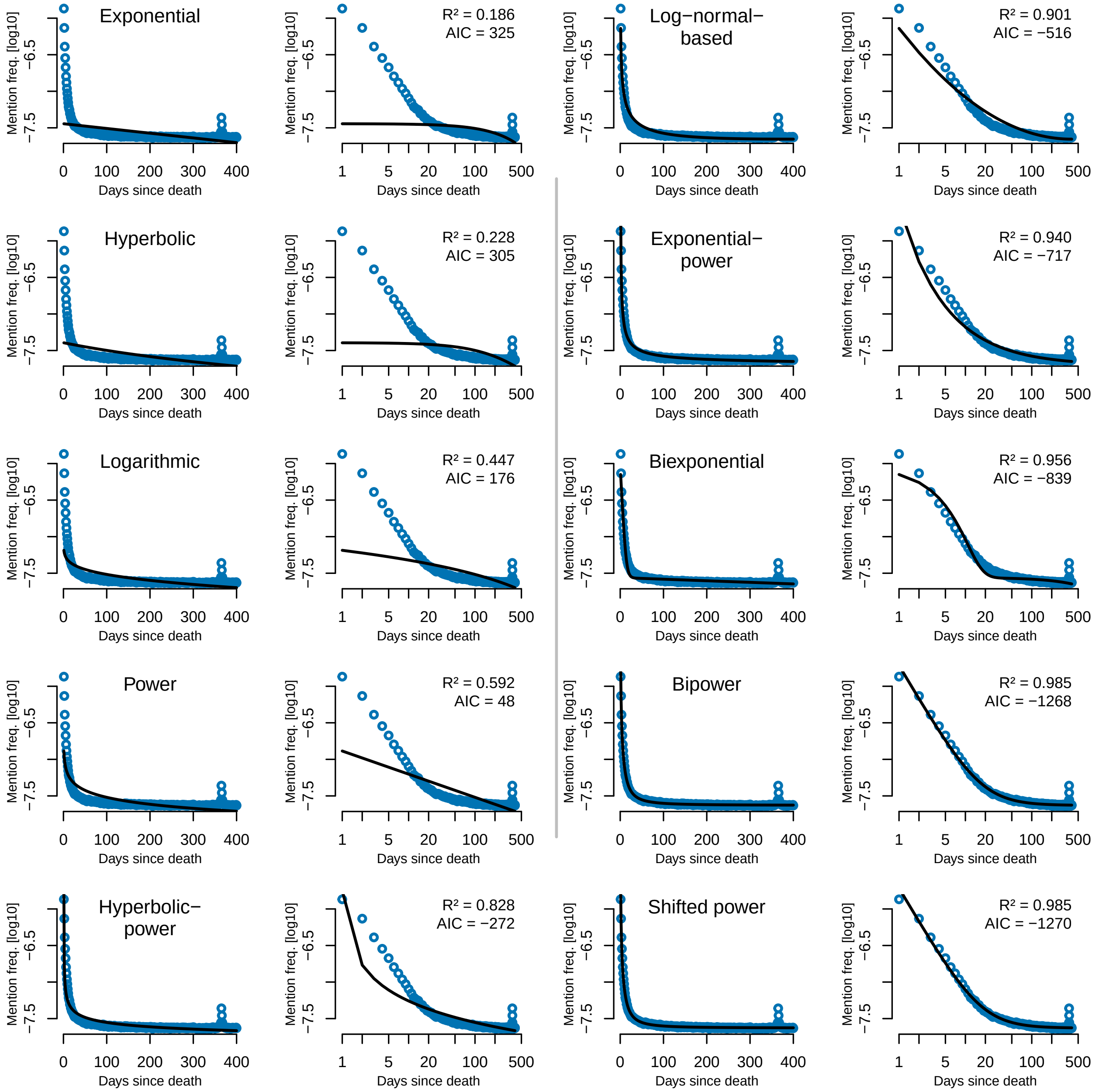

Supplementary Figure 2: Comparison of ten models $S(t)$ for fitting empirical mention frequencies, for **(a)** the news and **(b)** Twitter. All y-axes are logarithmic. The left and right plots in each pair show the same fits, the only difference being that the left plots have linear x-axes, whereas the right plots have logarithmic x-axes. Fits are nonlinear least-squares fits obtained in log space, i.e., logarithms were taken of both the empirical data and the model $S(t)$ before performing the least-squares optimization (see equation 1 in the paper for the case of the shifted power function). The biexponential function was introduced by Candia et al. ("The universal decay of collective memory and attention." *Nature Human Behaviour.* 2019; 3(1):82-91) and is parameterized by $N, p, q, r > 0$, as follows:

- Biexponential: $S(t) = \frac{N}{p+r-q}\left((p-q)e^{-(p+r)t} + re^{-qt}\right)$.

Based on the biexponential function, we define the (novel) bipower function by replacing exponentials with powers:

- Bipower: $S(t) = \frac{N}{p+r-q}\left((p-q)t^{-(p+r)} + rt^{-q}\right)$.

By fixing $q = 0$ and defining $a = \frac{Np}{p+r}$, $b = p + r$, and $c = \frac{Nr}{p+r}$, we obtain the shifted power function as a special case of the bipower function:

- Shifted power: $S(t) = at^{-b} + c$.

Theoretical motivations for six of the seven remaining functions are given by Rubin and Wenzel ("One hundred years of forgetting: A quantitative description of retention." *Psychological Review.* 1996; 103(4):734-760). Four of these functions are parameterized by two parameters $a, b > 0$, as follows:

- Exponential: $S(t) = a\,e^{-bt}$, i.e., $\log S(t) = \log a - be^{\log t}$.
- Hyperbolic: $S(t) = (a + bt)^{-1}$, i.e., $\log S(t) = -\log(a + be^{\log t})$.
- Logarithmic: $S(t) = a - b\log t$, i.e., $\log S(t) = \log(a - b\log t)$.
- Power: $S(t) = a\,t^{-b}$, i.e., $\log S(t) = \log a - b\log t$.

The four above functions share the property of being concave (exponential, hyperbolic, logarithmic) or linear (power) when plotted on log-log axes (i.e., $\log S(t)$ is a concave function of $\log t$, cf. right columns), whereas the empirical curves are convex on log-log axes.

Rubin and Wenzel also proposed generalized versions of the exponential and hyperbolic functions, called exponential-power and hyperbolic-power functions, respectively, where t is replaced by the power t^c, where c is a third parameter. (Analogous generalized versions of the logarithmic and power functions are not necessary, as they can already be expressed by the plain logarithmic and power functions, since $b\log t^c = (bc)\log t$.) For $c > 0$, the exponential-power and hyperbolic-power functions, too, are concave in log-log space, but when allowing for $b, c < 0$, they can be made convex and are thus better suited for fitting the empirical data (note that, in the following specifications, we maintain $a, b, c > 0$, but replace b by $-b$, and c by $-c$):

- Exponential-power: $S(t) = a\,e^{bt^{-c}}$, i.e., $\log S(t) = \log a + be^{-c\log t}$.
- Hyperbolic-power: $S(t) = (a - bt^{-c})^{-1}$, i.e., $\log S(t) = -\log(a - be^{-c\log t})$.

Finally, as the last function, we consider what Candia et al. refer to as the "log-normal" function, defined as $S(t) = \exp(\log a - b\log t - c(\log t)^2)$. To recognize the fact that, although this function takes the functional form of the log-normal distribution, it is not actually used to describe a probability distribution here, we refer to the function as "log-normal-based". The log-normal-based function, too, is concave in log-log space, but can be made convex by replacing c by $-c$, i.e., $S(t) = \exp(\log a - b\log t + c(\log t)^2)$. Note, however, that this results in $S(t)$ being an increasing function of t as $t \to \infty$, unlike the empirical data and unlike what one would require from a sound theoretical model of collective memory. We hence constrain the parameters such that the fitted function is monotonically decreasing over the modeled time range (days 1 to $t_{\max} = 400$). Since the unconstrained function, when fitted to the empirical data, assumes a minimum at $1 < t < t_{\max}$, the monotonicity constraint is equivalent to requiring the minimum to occur at $t = t_{\max}$, which happens for $b = -2ct_{\max}$ and gives rise to the following model:

- Log-normal-based: $S(t) = \exp(\log a + c((\log t)^2 - 2t_{\max}\log t))$, i.e., $\log S(t) = \log a + c((\log t)^2 - 2t_{\max}\log t)$.

We quantify goodness of fit using two measures (results in the legends of the right plots): (1) via coefficients of determination (R^2; computed as the squared correlation between observed and predicted values on the log scale; larger is better); (2) in order to account for the varying model complexity (the models have between two and four parameters), via Akaike's information criterion (AIC; smaller is better). R^2 and AIC result in the same ordering of the 10 models, and the ordering is identical across the two media (news and Twitter). In the figure, the models are sorted, top-down, in increasing order of goodness of fit. The shifted power model provides the best fit according to both measures (R^2 and AIC) and for both media (news and Twitter), with $R^2 = 0.989$ for the news and $R^2 = 0.985$ for Twitter. The bipower model yields essentially the same fit as the shifted power model, which, as mentioned above, is a special case of the bipower model with $q = 0$ fixed (optimal bipower fit: $q = 0.0068$ for the news, $q = 0$ for Twitter).

Proportion of cultural vs. communicative memory

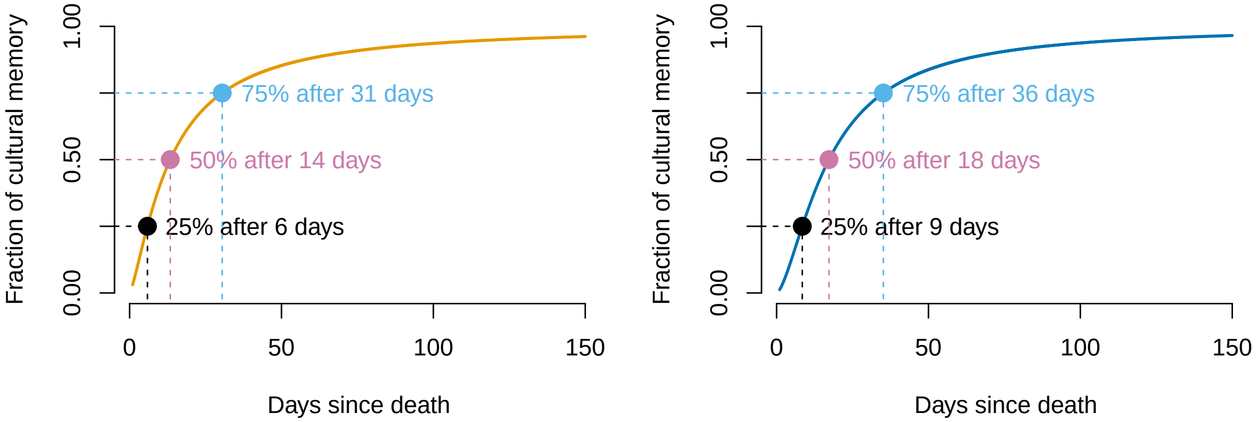

Supplementary Figure 3: Proportion of cultural vs. communicative memory. On day t after death, the total collective memory according to the shifted power law fit is $S(t) = u(t) + v(t)$, where $u(t) = at^{-b}$ is the communicative memory, and $v(t) = c$ is the cultural memory. We plot the proportion $v(t)/S(t)$ as a function of t. *Left:* news. *Right:* Twitter.

Document length

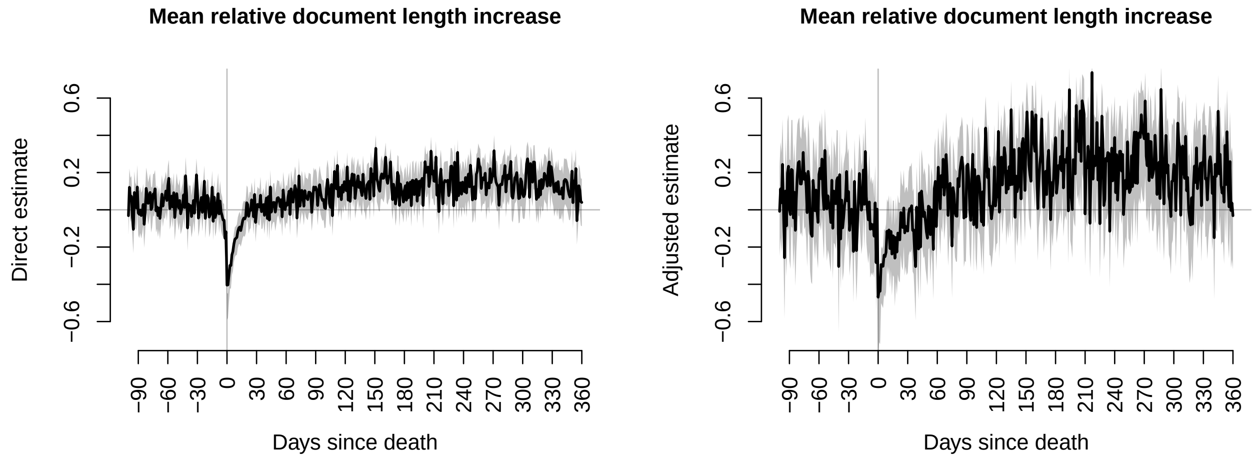

Supplementary Figure 4: Relative length increase of documents that mention deceased public figures (excluding Twitter posts), with respect to the respective public figure's pre-mortem mean document length, as a function of days since death. All means in this analysis are geometric means. Error bars are 95% confidence intervals approximated as ±2 standard errors. **Left:** Direct mean estimates of relative length increase. **Right:** Estimates adjusted for population drift (since certain groups of people are more likely to be mentioned post-mortem than others). Each day t's adjusted estimate was obtained from a separate linear regression model for day t only, which included each person i mentioned that day as a data point, with $\log(L_{it}/P_i)$ as the dependent variable (where L_{it} is i's mean document length on day t, and P_i is i's pre-mortem mean document length), and with independent variables defined by the predictors that were previously found to be significantly associated with short-term post-mortem mention frequency (language, pre-mortem mean mention frequency rank, manner of death, age group), such that estimates are for anglophones of median pre-mortem popularity who died an unnatural death at age 70–79 years. The adjusted estimate of the (geometric) mean relative length increase for day t is then given by $e^{a_t} - 1$, where a_t is the intercept of the regression for day t. We see that, both with and without adjustment, the documents that mentioned a person on the day of their death were on average about 40% shorter than documents that

mentioned the person before their death, and that the pre-mortem level is reached again (and then surpassed) after about one month.

Mention time series characteristics

Summaries of distributions of mention time series characteristics

(a) News

```
##  Pre-mortem mean  Short-term boost  Long-term boost      Halving time
##  Min.   :-5.851   Min.   :-0.2055   Min.   :-1.5490200   Min.   :0.01667
##  1st Qu.:-5.835   1st Qu.: 1.1924   1st Qu.:-0.0147554   1st Qu.:0.05903
##  Median :-5.819   Median : 1.9754   Median : 0.0005455   Median :0.16111
##  Mean   :-5.755   Mean   : 1.9171   Mean   : 0.0191381   Mean   :0.23172
##  3rd Qu.:-5.770   3rd Qu.: 2.6687   3rd Qu.: 0.0270671   3rd Qu.:0.35833
##  Max.   :-3.267   Max.   : 4.2103   Max.   : 1.3884812   Max.   :0.95833
```

(b) Twitter

```
##  Pre-mortem mean  Short-term boost  Long-term boost     Halving time
##  Min.   :-7.866   Min.   :-0.0413   Min.   :-0.557536   Min.   :0.01667
##  1st Qu.:-7.839   1st Qu.: 1.5856   1st Qu.:-0.006985   1st Qu.:0.06667
##  Median :-7.803   Median : 2.4474   Median : 0.016047   Median :0.15972
##  Mean   :-7.664   Mean   : 2.3262   Mean   : 0.055143   Mean   :0.22363
##  3rd Qu.:-7.671   3rd Qu.: 3.0998   3rd Qu.: 0.077694   3rd Qu.:0.33611
##  Max.   :-4.576   Max.   : 4.9904   Max.   : 1.552516   Max.   :0.88056
```

Supplementary Table 2: Summaries of distribution of mention time series characteristics for **(a)** the news and **(b)** Twitter.

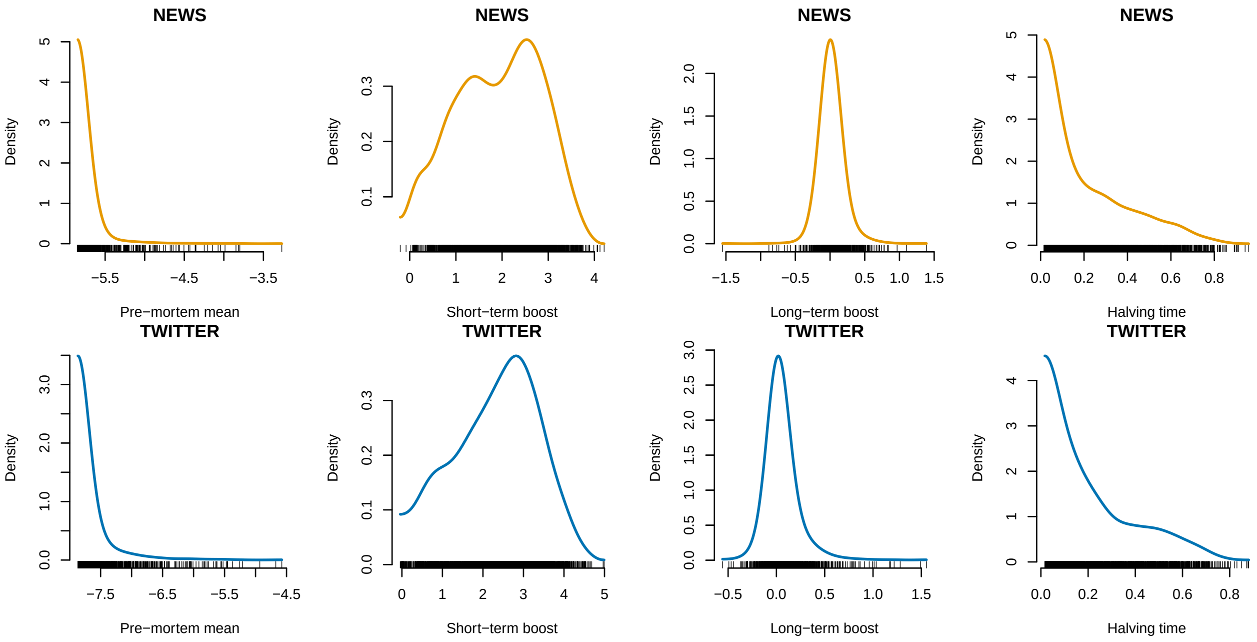

Supplementary Figure 5: Kernel-smoothed density plots of mention time series characteristics for the news (top) and Twitter (bottom).

Mention time series characteristics by notability type

(a) News

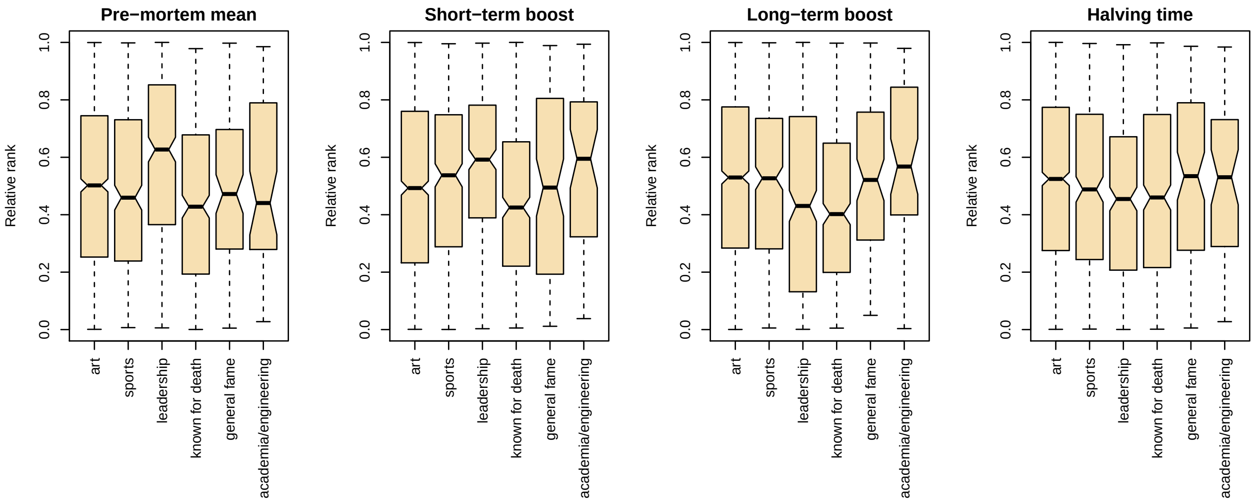

(b) Twitter

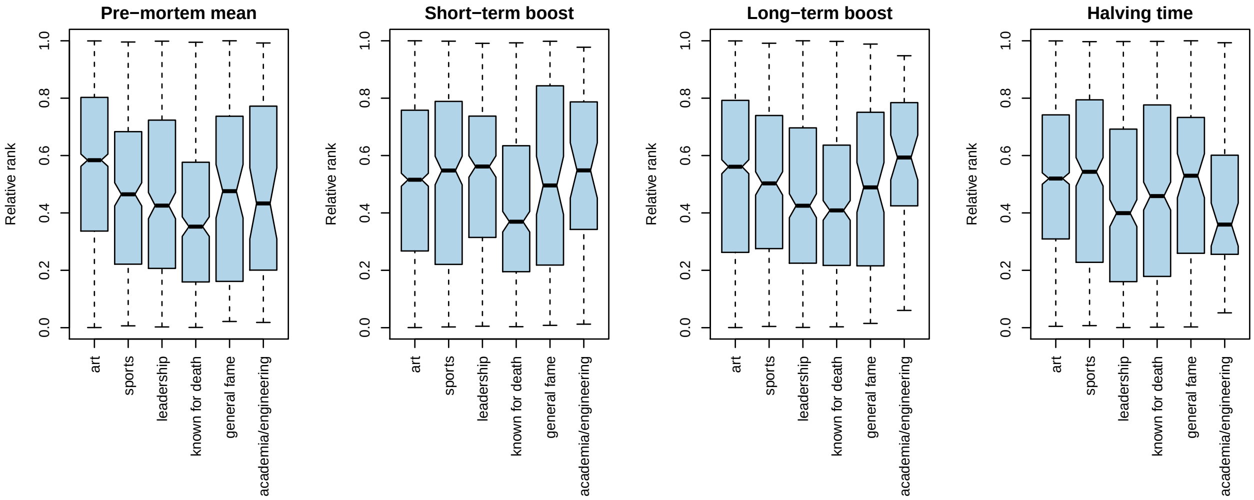

Supplementary Figure 6: Distributions of mention time series characteristics by notability type, visualized as box plots, for **(a)** the news and **(b)** Twitter. Boxes are bounded by the first and third quartiles; whiskers extend 1.5 inter-quartile ranges beyond the first and third quartiles (or to the minimum/maximum in case they fall within 1.5 inter-quartile ranges); the center bars mark medians, with notches corresponding to 95% confidence intervals of the median.

Cluster analysis

Optimal number of clusters

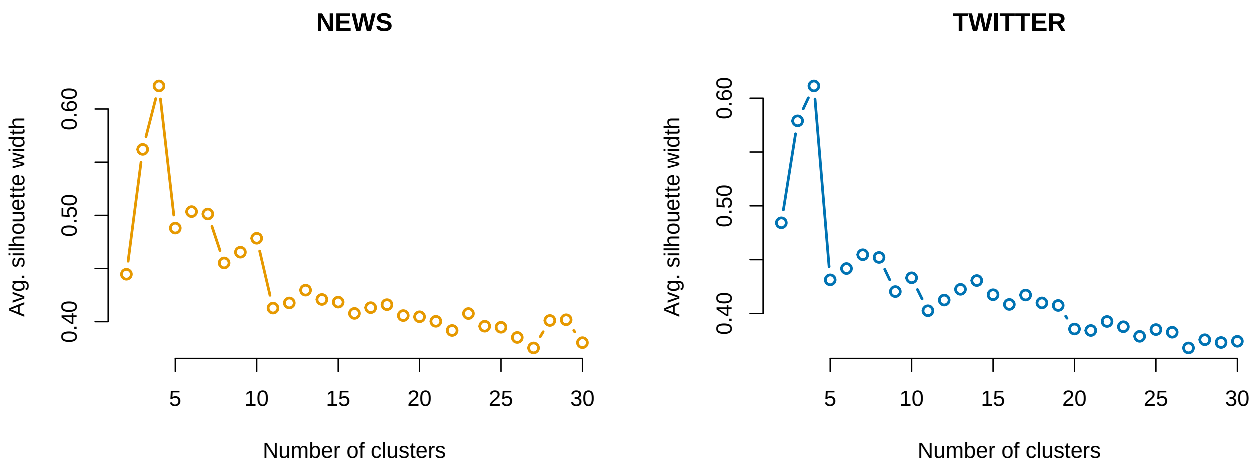

Supplementary Figure 7: Average silhouette width of clusterings produced by k-means algorithm, as a function of the number k of clusters (higher is better), for $k \in \{2, \ldots, 30\}$. Both **(a)** in the news and **(b)** on Twitter, $k = 4$ is optimal.

Overlay of time series per cluster

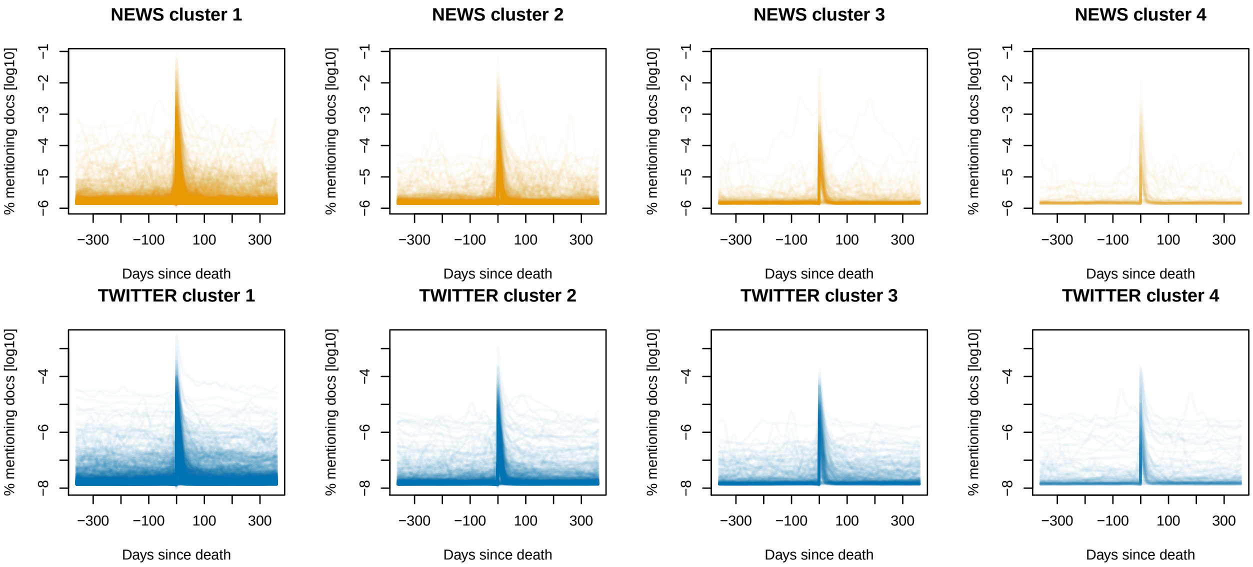

Supplementary Figure 8: Overlay of time series in each cluster, for news (top) and Twitter (bottom).

Regression modeling

Note: the variable names in the code may differ from those in the paper; see mapping in lists below.

Independent variables:

- pre-mortem mean (`mean_before`)
- age at death (`age_group`)

- manner of death (`death_type`)
- notability type (`type_group`)
- language (`anglo`)
- gender (`gender`)

Dependendent variables:

- short-term boost (`peak_mean_boost`)
- long-term boost (`perm_boost`)

We report results for two variants of each model, which differ in the way dependent variables are treated. Model variants are marked via the "Transformation on dependent variable" descriptor in the header of each model. The two variants are the following:

- `NONE`: dependent variables were used as-is. This variant is used in the main paper.
- `RELATIVE RANKS`: dependent variables were transformed to relative ranks; i.e., they were rank-transformed and then shifted/scaled to the interval $[-0.5, 0.5]$. This way, dependent variables need to be interpreted in relative terms: a value of 0 corresponds to the median, and positive [negative] values to ranks above [below] the median.

Models without interactions

```
##
##
## #####################################################
## REGRESSION MODEL
## Medium: NEWS
## Dependent variable: short-term boost
## Transformation on dependent variable: NONE
## #####################################################
##
## Call:
## lm(formula = as.formula(sprintf("peak_mean_boost%s ~ %s", suffix,
##     predictors)), data = lmdata)
##
## Residuals:
##     Min      1Q  Median      3Q     Max
## -2.3921 -0.4654  0.1215  0.5730  2.0660
##
## Coefficients:
##                               Estimate Std. Error t value Pr(>|t|)
## (Intercept)                    2.32157    0.06293  36.890  < 2e-16 ***
## mean_before_relrank            0.80417    0.09297   8.650  < 2e-16 ***
## age_group20                    0.16165    0.17015   0.950   0.3423
## age_group30                    0.40001    0.16740   2.390   0.0171 *
## age_group40                   -0.04607    0.12599  -0.366   0.7147
## age_group50                   -0.07459    0.09893  -0.754   0.4511
## age_group60                   -0.10927    0.08172  -1.337   0.1816
## age_group80                    0.02162    0.07826   0.276   0.7824
## age_group90                    0.17414    0.09779   1.781   0.0753 .
## death_typeunnatural            0.61845    0.09460   6.537 1.08e-10 ***
## genderFemale                   0.08287    0.07213   1.149   0.2509
## anglonon_anglo                -0.31567    0.07378  -4.279 2.09e-05 ***
## anglounknown                  -0.44551    0.08550  -5.211 2.36e-07 ***
## type_groupacademia/engineering  0.18120    0.19745   0.918   0.3590
```

```
## type_groupgeneral fame          0.06993    0.12391   0.564   0.5727
## type_groupknown for death      -0.10659    0.09933  -1.073   0.2835
## type_groupleadership            0.15217    0.08285   1.837   0.0666 .
## type_groupsports                0.04886    0.08336   0.586   0.5580
## ---
## Signif. codes:  0 '***' 0.001 '**' 0.01 '*' 0.05 '.' 0.1 ' ' 1
##
## Residual standard error: 0.7725 on 852 degrees of freedom
## Multiple R-squared:  0.2131, Adjusted R-squared:  0.1974
## F-statistic: 13.57 on 17 and 852 DF,  p-value: < 2.2e-16
##
##
##
## #####################################################
## REGRESSION MODEL
## Medium: NEWS
## Dependent variable: long-term boost
## Transformation on dependent variable: NONE
## #####################################################
##
## Call:
## lm(formula = as.formula(sprintf("perm_boost%s ~ %s", suffix,
##     predictors)), data = lmdata)
##
## Residuals:
##      Min       1Q   Median       3Q      Max
## -0.92517 -0.07187 -0.02152  0.03771  1.19435
##
## Coefficients:
##                                 Estimate Std. Error t value Pr(>|t|)
## (Intercept)                    0.0879028  0.0137837   6.377 2.95e-10 ***
## mean_before_relrank            0.0305324  0.0203625   1.499  0.13413
## age_group20                    0.0603247  0.0372663   1.619  0.10587
## age_group30                    0.0277988  0.0366633   0.758  0.44853
## age_group40                   -0.0009074  0.0275939  -0.033  0.97377
## age_group50                   -0.0578543  0.0216672  -2.670  0.00773 **
## age_group60                   -0.0499194  0.0178988  -2.789  0.00541 **
## age_group80                   -0.0181927  0.0171416  -1.061  0.28884
## age_group90                   -0.0111474  0.0214176  -0.520  0.60287
## death_typeunnatural            0.0973507  0.0207195   4.699 3.05e-06 ***
## genderFemale                   0.0343766  0.0157970   2.176  0.02982 *
## anglonon_anglo                -0.0610353  0.0161588  -3.777  0.00017 ***
## anglounknown                  -0.0790566  0.0187267  -4.222 2.69e-05 ***
## type_groupacademia/engineering -0.0315605  0.0432446  -0.730  0.46570
## type_groupgeneral fame        -0.0102523  0.0271399  -0.378  0.70571
## type_groupknown for death     -0.0213581  0.0217556  -0.982  0.32651
## type_groupleadership          -0.0581051  0.0181453  -3.202  0.00141 **
## type_groupsports              -0.0341565  0.0182585  -1.871  0.06173 .
## ---
## Signif. codes:  0 '***' 0.001 '**' 0.01 '*' 0.05 '.' 0.1 ' ' 1
##
## Residual standard error: 0.1692 on 852 degrees of freedom
## Multiple R-squared:  0.1234, Adjusted R-squared:  0.1059
## F-statistic: 7.056 on 17 and 852 DF,  p-value: 3.001e-16
```

```
##
##
##
## ####################################################
## REGRESSION MODEL
## Medium: TWITTER
## Dependent variable: short-term boost
## Transformation on dependent variable: NONE
## ####################################################
##
## Call:
## lm(formula = as.formula(sprintf("peak_mean_boost%s ~ %s", suffix,
##     predictors)), data = lmdata)
##
## Residuals:
##      Min       1Q   Median       3Q      Max
## -2.97464 -0.40997  0.07498  0.57097  2.10379
##
## Coefficients:
##                               Estimate Std. Error t value Pr(>|t|)
## (Intercept)                    2.67046    0.06666  40.061  < 2e-16 ***
## mean_before_relrank            0.94776    0.09955   9.521  < 2e-16 ***
## age_group20                    0.71833    0.17951   4.002 6.84e-05 ***
## age_group30                    0.64917    0.17658   3.676 0.000251 ***
## age_group40                    0.35056    0.13278   2.640 0.008438 **
## age_group50                    0.18143    0.10435   1.739 0.082470 .
## age_group60                    0.13015    0.08611   1.511 0.131051
## age_group80                    0.02061    0.08247   0.250 0.802727
## age_group90                    0.03437    0.10319   0.333 0.739172
## death_typeunnatural            0.28194    0.09978   2.826 0.004829 **
## genderFemale                  -0.03354    0.07591  -0.442 0.658720
## anglonon_anglo                -0.11627    0.07763  -1.498 0.134593
## anglounknown                  -0.32516    0.09079  -3.582 0.000361 ***
## type_groupacademia/engineering  0.33965    0.20822   1.631 0.103220
## type_groupgeneral fame         0.13243    0.13075   1.013 0.311418
## type_groupknown for death     -0.08814    0.10562  -0.835 0.404208
## type_groupleadership           0.11290    0.08677   1.301 0.193592
## type_groupsports               0.07169    0.08830   0.812 0.417031
## ---
## Signif. codes:  0 '***' 0.001 '**' 0.01 '*' 0.05 '.' 0.1 ' ' 1
##
## Residual standard error: 0.8148 on 852 degrees of freedom
## Multiple R-squared:  0.1917, Adjusted R-squared:  0.1755
## F-statistic: 11.88 on 17 and 852 DF,  p-value: < 2.2e-16
##
##
##
## ####################################################
## REGRESSION MODEL
## Medium: TWITTER
## Dependent variable: long-term boost
## Transformation on dependent variable: NONE
## ####################################################
##
```

```
## Call:
## lm(formula = as.formula(sprintf("perm_boost%s ~ %s", suffix,
##     predictors)), data = lmdata)
##
## Residuals:
##      Min       1Q   Median       3Q      Max
## -0.55367 -0.08110 -0.01437  0.05324  1.17998
##
## Coefficients:
##                                Estimate Std. Error t value Pr(>|t|)
## (Intercept)                    0.095062   0.014816   6.416 2.32e-10 ***
## mean_before_relrank            0.086441   0.022127   3.907 0.000101 ***
## age_group20                    0.192254   0.039899   4.818 1.71e-06 ***
## age_group30                    0.118044   0.039248   3.008 0.002711 **
## age_group40                    0.099524   0.029512   3.372 0.000779 ***
## age_group50                   -0.024487   0.023195  -1.056 0.291393
## age_group60                   -0.024520   0.019140  -1.281 0.200516
## age_group80                   -0.012644   0.018330  -0.690 0.490506
## age_group90                   -0.023678   0.022936  -1.032 0.302203
## death_typeunnatural            0.106270   0.022178   4.792 1.95e-06 ***
## genderFemale                   0.005622   0.016873   0.333 0.739083
## anglonon_anglo                -0.037016   0.017256  -2.145 0.032225 *
## anglounknown                  -0.080837   0.020179  -4.006 6.72e-05 ***
## type_groupacademia/engineering  0.023237   0.046282   0.502 0.615747
## type_groupgeneral fame        -0.007989   0.029062  -0.275 0.783457
## type_groupknown for death      0.008185   0.023476   0.349 0.727436
## type_groupleadership          -0.039897   0.019287  -2.069 0.038885 *
## type_groupsports              -0.034255   0.019626  -1.745 0.081269 .
## ---
## Signif. codes:  0 '***' 0.001 '**' 0.01 '*' 0.05 '.' 0.1 ' ' 1
##
## Residual standard error: 0.1811 on 852 degrees of freedom
## Multiple R-squared:  0.1776, Adjusted R-squared:  0.1612
## F-statistic: 10.82 on 17 and 852 DF,  p-value: < 2.2e-16
##
##
##
## #####################################################
## REGRESSION MODEL
## Medium: NEWS
## Dependent variable: short-term boost
## Transformation on dependent variable: RELATIVE RANKS
## #####################################################
##
## Call:
## lm(formula = as.formula(sprintf("peak_mean_boost%s ~ %s", suffix,
##     predictors)), data = lmdata)
##
## Residuals:
##      Min       1Q   Median       3Q      Max
## -0.62044 -0.19422  0.01128  0.20580  0.74237
##
## Coefficients:
##                                Estimate Std. Error t value Pr(>|t|)
```

```
## (Intercept)                     0.001595   0.020820   0.077   0.9390
## mean_before_relrank             0.292185   0.030757   9.500  < 2e-16 ***
## age_group20                     0.054393   0.056290   0.966   0.3342
## age_group30                     0.119792   0.055379   2.163   0.0308 *
## age_group40                    -0.020965   0.041680  -0.503   0.6151
## age_group50                    -0.029145   0.032728  -0.891   0.3734
## age_group60                    -0.046225   0.027036  -1.710   0.0877 .
## age_group80                     0.011330   0.025892   0.438   0.6618
## age_group90                     0.070412   0.032351   2.177   0.0298 *
## death_typeunnatural             0.223393   0.031296   7.138 2.03e-12 ***
## genderFemale                    0.032258   0.023861   1.352   0.1768
## anglonon_anglo                 -0.100770   0.024407  -4.129 4.01e-05 ***
## anglounknown                   -0.149597   0.028286  -5.289 1.57e-07 ***
## type_groupacademia/engineering  0.054180   0.065320   0.829   0.4071
## type_groupgeneral fame          0.025013   0.040994   0.610   0.5419
## type_groupknown for death      -0.041843   0.032861  -1.273   0.2033
## type_groupleadership            0.027634   0.027408   1.008   0.3136
## type_groupsports                0.001862   0.027579   0.068   0.9462
## ---
## Signif. codes:  0 '***' 0.001 '**' 0.01 '*' 0.05 '.' 0.1 ' ' 1
##
## Residual standard error: 0.2555 on 852 degrees of freedom
## Multiple R-squared:  0.2343, Adjusted R-squared:  0.219
## F-statistic: 15.34 on 17 and 852 DF,  p-value: < 2.2e-16
##
##
##
## #####################################################
## REGRESSION MODEL
## Medium: NEWS
## Dependent variable: long-term boost
## Transformation on dependent variable: RELATIVE RANKS
## #####################################################
##
## Call:
## lm(formula = as.formula(sprintf("perm_boost%s ~ %s", suffix,
##     predictors)), data = lmdata)
##
## Residuals:
##      Min       1Q   Median       3Q      Max
## -0.60548 -0.21923  0.00579  0.23231  0.57350
##
## Coefficients:
##                                 Estimate Std. Error t value Pr(>|t|)
## (Intercept)                     0.069386   0.022386   3.099 0.002002 **
## mean_before_relrank            -0.035601   0.033071  -1.076 0.282009
## age_group20                     0.014011   0.060525   0.231 0.816994
## age_group30                    -0.025187   0.059546  -0.423 0.672407
## age_group40                    -0.043987   0.044816  -0.982 0.326618
## age_group50                    -0.116348   0.035190  -3.306 0.000985 ***
## age_group60                    -0.085145   0.029070  -2.929 0.003491 **
## age_group80                    -0.009021   0.027840  -0.324 0.745990
## age_group90                     0.039381   0.034785   1.132 0.257902
## death_typeunnatural             0.134966   0.033651   4.011 6.58e-05 ***
```

```
## genderFemale                      0.037946   0.025656   1.479 0.139504
## anglonon_anglo                   -0.108631   0.026244  -4.139 3.83e-05 ***
## anglounknown                     -0.145758   0.030415  -4.792 1.94e-06 ***
## type_groupacademia/engineering -0.019779   0.070235  -0.282 0.778306
## type_groupgeneral fame           -0.002759   0.044079  -0.063 0.950104
## type_groupknown for death        -0.064889   0.035334  -1.836 0.066638 .
## type_groupleadership             -0.071294   0.029470  -2.419 0.015763 *
## type_groupsports                 -0.042940   0.029654  -1.448 0.147977
## ---
## Signif. codes:  0 '***' 0.001 '**' 0.01 '*' 0.05 '.' 0.1 ' ' 1
##
## Residual standard error: 0.2748 on 852 degrees of freedom
## Multiple R-squared:  0.1148, Adjusted R-squared:  0.0971
## F-statistic: 6.498 on 17 and 852 DF,  p-value: 1.155e-14
##
##
##
## #####################################################
## REGRESSION MODEL
## Medium: TWITTER
## Dependent variable: short-term boost
## Transformation on dependent variable: RELATIVE RANKS
## #####################################################
##
## Call:
## lm(formula = as.formula(sprintf("peak_mean_boost%s ~ %s", suffix,
##     predictors)), data = lmdata)
##
## Residuals:
##      Min       1Q   Median       3Q      Max
## -0.71673 -0.18473 -0.00208  0.20710  0.61854
##
## Coefficients:
##                                 Estimate Std. Error t value Pr(>|t|)
## (Intercept)                      -0.033516   0.021153  -1.584 0.113459
## mean_before_relrank               0.336775   0.031589  10.661  < 2e-16 ***
## age_group20                       0.224402   0.056963   3.939 8.84e-05 ***
## age_group30                       0.216619   0.056034   3.866 0.000119 ***
## age_group40                       0.099944   0.042134   2.372 0.017910 *
## age_group50                       0.048430   0.033115   1.462 0.143973
## age_group60                       0.038284   0.027326   1.401 0.161574
## age_group80                       0.003695   0.026169   0.141 0.887740
## age_group90                       0.006365   0.032745   0.194 0.845930
## death_typeunnatural               0.095068   0.031662   3.003 0.002756 **
## genderFemale                     -0.007467   0.024089  -0.310 0.756643
## anglonon_anglo                   -0.030620   0.024636  -1.243 0.214240
## anglounknown                     -0.107934   0.028809  -3.746 0.000191 ***
## type_groupacademia/engineering  0.120612   0.066075   1.825 0.068295 .
## type_groupgeneral fame            0.052868   0.041492   1.274 0.202944
## type_groupknown for death        -0.026970   0.033516  -0.805 0.421209
## type_groupleadership              0.025037   0.027535   0.909 0.363470
## type_groupsports                  0.026369   0.028019   0.941 0.346917
## ---
## Signif. codes:  0 '***' 0.001 '**' 0.01 '*' 0.05 '.' 0.1 ' ' 1
```

```
##
## Residual standard error: 0.2586 on 852 degrees of freedom
## Multiple R-squared:  0.2161, Adjusted R-squared:  0.2004
## F-statistic: 13.82 on 17 and 852 DF,  p-value: < 2.2e-16
##
##
##
## ####################################################
## REGRESSION MODEL
## Medium: TWITTER
## Dependent variable: long-term boost
## Transformation on dependent variable: RELATIVE RANKS
## ####################################################
##
## Call:
## lm(formula = as.formula(sprintf("perm_boost%s ~ %s", suffix,
##     predictors)), data = lmdata)
##
## Residuals:
##      Min       1Q   Median       3Q      Max
## -0.67278 -0.19342  0.02505  0.21969  0.60132
##
## Coefficients:
##                                Estimate Std. Error t value Pr(>|t|)
## (Intercept)                    0.038607   0.022426   1.722 0.085511 .
## mean_before_relrank            0.118846   0.033490   3.549 0.000408 ***
## age_group20                    0.111425   0.060390   1.845 0.065372 .
## age_group30                    0.106071   0.059405   1.786 0.074526 .
## age_group40                    0.060312   0.044669   1.350 0.177307
## age_group50                   -0.060151   0.035107  -1.713 0.087008 .
## age_group60                   -0.044402   0.028970  -1.533 0.125726
## age_group80                   -0.015164   0.027744  -0.547 0.584804
## age_group90                   -0.033081   0.034715  -0.953 0.340897
## death_typeunnatural            0.096865   0.033567   2.886 0.004004 **
## genderFemale                   0.029224   0.025538   1.144 0.252806
## anglonon_anglo                -0.031642   0.026118  -1.212 0.226027
## anglounknown                  -0.139591   0.030542  -4.570 5.59e-06 ***
## type_groupacademia/engineering  0.076049   0.070050   1.086 0.277952
## type_groupgeneral fame         0.006527   0.043988   0.148 0.882072
## type_groupknown for death     -0.025012   0.035532  -0.704 0.481664
## type_groupleadership          -0.102206   0.029192  -3.501 0.000487 ***
## type_groupsports              -0.025534   0.029704  -0.860 0.390257
## ---
## Signif. codes:  0 '***' 0.001 '**' 0.01 '*' 0.05 '.' 0.1 ' ' 1
##
## Residual standard error: 0.2741 on 852 degrees of freedom
## Multiple R-squared:  0.1189, Adjusted R-squared:  0.1013
## F-statistic: 6.765 on 17 and 852 DF,  p-value: 2.014e-15
```

Models with "age by manner of death" interaction

```
##
##
```

```
## ####################################################
## REGRESSION MODEL
## Medium: NEWS
## Dependent variable: short-term boost
## Transformation on dependent variable: NONE
## ####################################################
##
## Call:
## lm(formula = as.formula(sprintf("peak_mean_boost%s ~ %s", suffix,
##     predictors)), data = lmdata)
##
## Residuals:
##     Min      1Q  Median      3Q     Max
## -2.3913 -0.4656  0.1301  0.5624  2.0282
##
## Coefficients:
##                                Estimate Std. Error t value Pr(>|t|)
## mean_before_relrank             0.79350    0.09382   8.458  < 2e-16 ***
## age_group70                     2.31291    0.06392  36.184  < 2e-16 ***
## age_group20                     2.70452    0.26667  10.142  < 2e-16 ***
## age_group30                     2.62259    0.23921  10.964  < 2e-16 ***
## age_group40                     2.22989    0.13600  16.397  < 2e-16 ***
## age_group50                     2.28254    0.09768  23.368  < 2e-16 ***
## age_group60                     2.21414    0.06950  31.859  < 2e-16 ***
## age_group80                     2.34947    0.06286  37.377  < 2e-16 ***
## age_group90                     2.50226    0.08461  29.574  < 2e-16 ***
## death_typeunnatural             0.93213    0.30056   3.101  0.00199 **
## genderFemale                    0.08619    0.07254   1.188  0.23513
## anglonon_anglo                 -0.32918    0.07476  -4.403 1.20e-05 ***
## anglounknown                   -0.45736    0.08604  -5.316 1.36e-07 ***
## type_groupacademia/engineering  0.17904    0.19785   0.905  0.36577
## type_groupgeneral fame          0.05273    0.12475   0.423  0.67264
## type_groupknown for death      -0.09979    0.09996  -0.998  0.31843
## type_groupleadership            0.15945    0.08318   1.917  0.05559 .
## type_groupsports                0.04115    0.08387   0.491  0.62376
## age_group20:death_typeunnatural -0.63094    0.43567  -1.448  0.14793
## age_group30:death_typeunnatural -0.14156    0.42659  -0.332  0.74009
## age_group40:death_typeunnatural -0.16313    0.37877  -0.431  0.66682
## age_group50:death_typeunnatural -0.44845    0.35775  -1.254  0.21037
## age_group60:death_typeunnatural -0.30088    0.35364  -0.851  0.39511
## age_group80:death_typeunnatural -0.47493    0.54408  -0.873  0.38296
## age_group90:death_typeunnatural -0.81402    0.83740  -0.972  0.33129
## ---
## Signif. codes:  0 '***' 0.001 '**' 0.01 '*' 0.05 '.' 0.1 ' ' 1
##
## Residual standard error: 0.7739 on 845 degrees of freedom
## Multiple R-squared:  0.9055, Adjusted R-squared:  0.9027
## F-statistic: 323.8 on 25 and 845 DF,  p-value: < 2.2e-16
##
##
##
## ####################################################
## REGRESSION MODEL
## Medium: NEWS
```

```
## Dependent variable: long-term boost
## Transformation on dependent variable: NONE
## ######################################################
##
## Call:
## lm(formula = as.formula(sprintf("perm_boost%s ~ %s", suffix,
##     predictors)), data = lmdata)
##
## Residuals:
##      Min       1Q   Median       3Q      Max
## -0.92547 -0.07094 -0.02103  0.03979  1.15319
##
## Coefficients:
##                                Estimate Std. Error t value Pr(>|t|)
## mean_before_relrank             0.025414   0.020412   1.245 0.213448
## age_group70                     0.089665   0.013907   6.448 1.91e-10 ***
## age_group20                     0.060730   0.058016   1.047 0.295500
## age_group30                     0.055130   0.052042   1.059 0.289748
## age_group40                     0.067078   0.029587   2.267 0.023633 *
## age_group50                     0.053144   0.021251   2.501 0.012580 *
## age_group60                     0.037372   0.015120   2.472 0.013643 *
## age_group80                     0.070787   0.013675   5.176 2.83e-07 ***
## age_group90                     0.078577   0.018407   4.269 2.19e-05 ***
## death_typeunnatural             0.054179   0.065388   0.829 0.407578
## genderFemale                    0.035626   0.015782   2.257 0.024240 *
## anglonon_anglo                 -0.061710   0.016264  -3.794 0.000159 ***
## anglounknown                   -0.080713   0.018718  -4.312 1.81e-05 ***
## type_groupacademia/engineering -0.032227   0.043044  -0.749 0.454248
## type_groupgeneral fame         -0.016796   0.027139  -0.619 0.536159
## type_groupknown for death      -0.024095   0.021748  -1.108 0.268199
## type_groupleadership           -0.057120   0.018097  -3.156 0.001654 **
## type_groupsports               -0.032155   0.018246  -1.762 0.078382 .
## age_group20:death_typeunnatural  0.171955   0.094784   1.814 0.070003 .
## age_group30:death_typeunnatural  0.144053   0.092807   1.552 0.120995
## age_group40:death_typeunnatural  0.103868   0.082405   1.260 0.207855
## age_group50:death_typeunnatural -0.060255   0.077832  -0.774 0.439047
## age_group60:death_typeunnatural  0.053030   0.076937   0.689 0.490847
## age_group80:death_typeunnatural  0.009704   0.118368   0.082 0.934680
## age_group90:death_typeunnatural -0.144978   0.182183  -0.796 0.426381
## ---
## Signif. codes:  0 '***' 0.001 '**' 0.01 '*' 0.05 '.' 0.1 ' ' 1
##
## Residual standard error: 0.1684 on 845 degrees of freedom
## Multiple R-squared:  0.1994, Adjusted R-squared:  0.1757
## F-statistic: 8.418 on 25 and 845 DF,  p-value: < 2.2e-16

##
##
## ######################################################
## REGRESSION MODEL
## Medium: TWITTER
## Dependent variable: short-term boost
## Transformation on dependent variable: NONE
## ######################################################
```

```
##
## Call:
## lm(formula = as.formula(sprintf("peak_mean_boost%s ~ %s", suffix,
##     predictors)), data = lmdata)
##
## Residuals:
##      Min       1Q   Median       3Q      Max
## -2.94216 -0.41102  0.07389  0.57059  2.18694
##
## Coefficients:
##                                Estimate Std. Error t value Pr(>|t|)
## mean_before_relrank             0.94026    0.10036   9.369  < 2e-16 ***
## age_group70                     2.66931    0.06775  39.397  < 2e-16 ***
## age_group20                     3.47492    0.28167  12.337  < 2e-16 ***
## age_group30                     3.29553    0.25261  13.046  < 2e-16 ***
## age_group40                     2.93793    0.14373  20.441  < 2e-16 ***
## age_group50                     2.87237    0.10330  27.806  < 2e-16 ***
## age_group60                     2.80662    0.07339  38.240  < 2e-16 ***
## age_group80                     2.70010    0.06692  40.350  < 2e-16 ***
## age_group90                     2.70831    0.08941  30.292  < 2e-16 ***
## death_typeunnatural             0.37666    0.31740   1.187 0.235683
## genderFemale                   -0.03440    0.07644  -0.450 0.652773
## anglonon_anglo                 -0.12447    0.07878  -1.580 0.114517
## anglounknown                   -0.33028    0.09150  -3.610 0.000325 ***
## type_groupacademia/engineering  0.33479    0.20886   1.603 0.109313
## type_groupgeneral fame          0.11715    0.13179   0.889 0.374306
## type_groupknown for death      -0.08548    0.10636  -0.804 0.421793
## type_groupleadership            0.11857    0.08718   1.360 0.174159
## type_groupsports                0.06552    0.08892   0.737 0.461390
## age_group20:death_typeunnatural -0.21410    0.46007  -0.465 0.641789
## age_group30:death_typeunnatural -0.04720    0.45020  -0.105 0.916533
## age_group40:death_typeunnatural  0.16610    0.39898   0.416 0.677281
## age_group50:death_typeunnatural -0.16983    0.37827  -0.449 0.653564
## age_group60:death_typeunnatural -0.12923    0.37346  -0.346 0.729391
## age_group80:death_typeunnatural -0.49657    0.57411  -0.865 0.387321
## age_group90:death_typeunnatural -0.24853    0.88473  -0.281 0.778846
## ---
## Signif. codes:  0 '***' 0.001 '**' 0.01 '*' 0.05 '.' 0.1 ' ' 1
##
## Residual standard error: 0.8171 on 845 degrees of freedom
## Multiple R-squared:  0.9241, Adjusted R-squared:  0.9218
## F-statistic: 411.4 on 25 and 845 DF,  p-value: < 2.2e-16
##
##
##
## ####################################################
## REGRESSION MODEL
## Medium: TWITTER
## Dependent variable: long-term boost
## Transformation on dependent variable: NONE
## ####################################################
##
## Call:
## lm(formula = as.formula(sprintf("perm_boost%s ~ %s", suffix,
```

```
##     predictors)), data = lmdata)
##
## Residuals:
##      Min       1Q   Median       3Q      Max
## -0.64801 -0.07463 -0.01514  0.04831  1.08654
##
## Coefficients:
##                                  Estimate Std. Error t value Pr(>|t|)
## mean_before_relrank              0.083630   0.022064   3.790 0.000161 ***
## age_group70                      0.094879   0.014896   6.369 3.11e-10 ***
## age_group20                      0.079564   0.061926   1.285 0.199202
## age_group30                      0.207745   0.055537   3.741 0.000196 ***
## age_group40                      0.196045   0.031600   6.204 8.61e-10 ***
## age_group50                      0.086549   0.022711   3.811 0.000148 ***
## age_group60                      0.071046   0.016136   4.403 1.21e-05 ***
## age_group80                      0.082052   0.014712   5.577 3.29e-08 ***
## age_group90                      0.072244   0.019656   3.675 0.000253 ***
## death_typeunnatural              0.090447   0.069782   1.296 0.195279
## genderFemale                     0.006957   0.016805   0.414 0.678970
## anglonon_anglo                  -0.036397   0.017321  -2.101 0.035909 *
## anglounknown                    -0.079850   0.020116  -3.969 7.82e-05 ***
## type_groupacademia/engineering   0.022898   0.045918   0.499 0.618139
## type_groupgeneral fame          -0.011641   0.028974  -0.402 0.687945
## type_groupknown for death        0.006300   0.023383   0.269 0.787670
## type_groupleadership            -0.040311   0.019166  -2.103 0.035737 *
## type_groupsports                -0.028105   0.019549  -1.438 0.150895
## age_group20:death_typeunnatural  0.318038   0.101148   3.144 0.001723 **
## age_group30:death_typeunnatural  0.021538   0.098979   0.218 0.827789
## age_group40:death_typeunnatural  0.007598   0.087717   0.087 0.930992
## age_group50:death_typeunnatural -0.063518   0.083164  -0.764 0.445219
## age_group60:death_typeunnatural  0.003213   0.082106   0.039 0.968796
## age_group80:death_typeunnatural  0.009910   0.126221   0.079 0.937438
## age_group90:death_typeunnatural -0.113872   0.194512  -0.585 0.558420
## ---
## Signif. codes:  0 '***' 0.001 '**' 0.01 '*' 0.05 '.' 0.1 ' ' 1
##
## Residual standard error: 0.1796 on 845 degrees of freedom
## Multiple R-squared:  0.3217, Adjusted R-squared:  0.3016
## F-statistic: 16.03 on 25 and 845 DF,  p-value: < 2.2e-16

##
##
## #####################################################
## REGRESSION MODEL
## Medium: NEWS
## Dependent variable: short-term boost
## Transformation on dependent variable: RELATIVE RANKS
## #####################################################
##
## Call:
## lm(formula = as.formula(sprintf("peak_mean_boost%s ~ %s", suffix,
##     predictors)), data = lmdata)
##
## Residuals:
```

```
##      Min       1Q   Median       3Q      Max 
## -0.62147 -0.19550  0.01088  0.20087  0.73057 
## 
## Coefficients:
##                                  Estimate Std. Error t value Pr(>|t|)    
## mean_before_relrank             0.2876073  0.0310288   9.269  < 2e-16 ***
## age_group70                    -0.0010522  0.0211400  -0.050  0.96031    
## age_group20                     0.1238746  0.0881930   1.405  0.16051    
## age_group30                     0.0812147  0.0791111   1.027  0.30491    
## age_group40                    -0.0379687  0.0449768  -0.844  0.39881    
## age_group50                    -0.0170764  0.0323044  -0.529  0.59722    
## age_group60                    -0.0432214  0.0229844  -1.880  0.06039 .  
## age_group80                     0.0149510  0.0207886   0.719  0.47222    
## age_group90                     0.0749740  0.0279819   2.679  0.00752 ** 
## death_typeunnatural             0.3175289  0.0993996   3.194  0.00145 ** 
## genderFemale                    0.0340646  0.0239914   1.420  0.15602    
## anglonon_anglo                 -0.1047908  0.0247235  -4.239  2.5e-05 ***
## anglounknown                   -0.1532241  0.0284546  -5.385  9.4e-08 ***
## type_groupacademia/engineering  0.0530173  0.0654330   0.810  0.41802    
## type_groupgeneral fame          0.0191595  0.0412557   0.464  0.64247    
## type_groupknown for death      -0.0403775  0.0330598  -1.221  0.22229    
## type_groupleadership            0.0299099  0.0275100   1.087  0.27724    
## type_groupsports               -0.0005107  0.0277366  -0.018  0.98531    
## age_group20:death_typeunnatural -0.1915827  0.1440851  -1.330  0.18399    
## age_group30:death_typeunnatural -0.0252587  0.1410809  -0.179  0.85795    
## age_group40:death_typeunnatural -0.0343547  0.1252681  -0.274  0.78396    
## age_group50:death_typeunnatural -0.1337441  0.1183163  -1.130  0.25863    
## age_group60:death_typeunnatural -0.0985255  0.1169552  -0.842  0.39979    
## age_group80:death_typeunnatural -0.1499235  0.1799365  -0.833  0.40497    
## age_group90:death_typeunnatural -0.3572365  0.2769449  -1.290  0.19743    
## ---
## Signif. codes:  0 '***' 0.001 '**' 0.01 '*' 0.05 '.' 0.1 ' ' 1
## 
## Residual standard error: 0.2559 on 845 degrees of freedom
## Multiple R-squared:  0.2383, Adjusted R-squared:  0.2157 
## F-statistic: 10.57 on 25 and 845 DF,  p-value: < 2.2e-16
## 
## 
## 
## #####################################################
## REGRESSION MODEL
## Medium: NEWS
## Dependent variable: long-term boost
## Transformation on dependent variable: RELATIVE RANKS
## #####################################################
## 
## Call:
## lm(formula = as.formula(sprintf("perm_boost%s ~ %s", suffix, 
##     predictors)), data = lmdata)
## 
## Residuals:
##      Min       1Q   Median       3Q      Max 
## -0.63192 -0.21951  0.00156  0.23031  0.57993 
## 
```

```
## Coefficients:
##                                  Estimate Std. Error t value Pr(>|t|)
## mean_before_relrank             -0.039227   0.033372  -1.175 0.240153
## age_group70                      0.070331   0.022736   3.093 0.002045 **
## age_group20                      0.096091   0.094853   1.013 0.311325
## age_group30                      0.041532   0.085085   0.488 0.625588
## age_group40                      0.014224   0.048373   0.294 0.768798
## age_group50                     -0.025681   0.034744  -0.739 0.460022
## age_group60                     -0.022031   0.024720  -0.891 0.373062
## age_group80                      0.062061   0.022358   2.776 0.005630 **
## age_group90                      0.112468   0.030095   3.737 0.000199 ***
## death_typeunnatural              0.147611   0.106906   1.381 0.167721
## genderFemale                     0.038463   0.025803   1.491 0.136437
## anglonon_anglo                  -0.113644   0.026591  -4.274 2.14e-05 ***
## anglounknown                    -0.149934   0.030603  -4.899 1.15e-06 ***
## type_groupacademia/engineering  -0.019377   0.070374  -0.275 0.783122
## type_groupgeneral fame          -0.008856   0.044371  -0.200 0.841847
## type_groupknown for death       -0.064415   0.035556  -1.812 0.070396 .
## type_groupleadership            -0.070072   0.029587  -2.368 0.018094 *
## type_groupsports                -0.044702   0.029831  -1.498 0.134378
## age_group20:death_typeunnatural -0.027778   0.154966  -0.179 0.857782
## age_group30:death_typeunnatural -0.004357   0.151735  -0.029 0.977101
## age_group40:death_typeunnatural  0.026451   0.134728   0.196 0.844400
## age_group50:death_typeunnatural -0.098592   0.127251  -0.775 0.438686
## age_group60:death_typeunnatural  0.059818   0.125787   0.476 0.634521
## age_group80:death_typeunnatural -0.009561   0.193525  -0.049 0.960608
## age_group90:death_typeunnatural -0.288201   0.297859  -0.968 0.333534
## ---
## Signif. codes:  0 '***' 0.001 '**' 0.01 '*' 0.05 '.' 0.1 ' ' 1
##
## Residual standard error: 0.2753 on 845 degrees of freedom
## Multiple R-squared:  0.1189, Adjusted R-squared:  0.09283
## F-statistic: 4.561 on 25 and 845 DF,  p-value: 2.333e-12

##
##
## #####################################################
## REGRESSION MODEL
## Medium: TWITTER
## Dependent variable: short-term boost
## Transformation on dependent variable: RELATIVE RANKS
## #####################################################
##
## Call:
## lm(formula = as.formula(sprintf("peak_mean_boost%s ~ %s", suffix,
##     predictors)), data = lmdata)
##
## Residuals:
##      Min       1Q   Median       3Q      Max
## -0.71524 -0.18611 -0.00282  0.20651  0.61822
##
## Coefficients:
##                                  Estimate Std. Error t value Pr(>|t|)
## mean_before_relrank              0.334205   0.031828  10.500  < 2e-16 ***
```

```
## age_group70                      -0.033041   0.021488  -1.538 0.124511
## age_group20                       0.237288   0.089330   2.656 0.008049 **
## age_group30                       0.175763   0.080115   2.194 0.028516 *
## age_group40                       0.035998   0.045584   0.790 0.429916
## age_group50                       0.020332   0.032761   0.621 0.535032
## age_group60                       0.005842   0.023277   0.251 0.801897
## age_group80                      -0.026348   0.021223  -1.241 0.214778
## age_group90                      -0.025258   0.028355  -0.891 0.373308
## death_typeunnatural               0.108666   0.100663   1.079 0.280675
## genderFemale                     -0.007697   0.024242  -0.318 0.750933
## anglonon_anglo                   -0.033544   0.024986  -1.342 0.179797
## anglounknown                     -0.109642   0.029019  -3.778 0.000169 ***
## type_groupacademia/engineering   0.118763   0.066238   1.793 0.073337 .
## type_groupgeneral fame            0.047796   0.041796   1.144 0.253129
## type_groupknown for death        -0.026487   0.033731  -0.785 0.432540
## type_groupleadership              0.026762   0.027648   0.968 0.333343
## type_groupsports                  0.023447   0.028200   0.831 0.405954
## age_group20:death_typeunnatural -0.078726   0.145910  -0.540 0.589650
## age_group30:death_typeunnatural  0.001729   0.142781   0.012 0.990343
## age_group40:death_typeunnatural  0.082247   0.126535   0.650 0.515874
## age_group50:death_typeunnatural -0.030965   0.119968  -0.258 0.796386
## age_group60:death_typeunnatural -0.014870   0.118442  -0.126 0.900118
## age_group80:death_typeunnatural -0.166931   0.182080  -0.917 0.359509
## age_group90:death_typeunnatural -0.125260   0.280592  -0.446 0.655414
## ---
## Signif. codes:  0 '***' 0.001 '**' 0.01 '*' 0.05 '.' 0.1 ' ' 1
##
## Residual standard error: 0.2591 on 845 degrees of freedom
## Multiple R-squared:  0.2191, Adjusted R-squared:  0.196
## F-statistic: 9.482 on 25 and 845 DF,  p-value: < 2.2e-16
##
##
##
## ####################################################
## REGRESSION MODEL
## Medium: TWITTER
## Dependent variable: long-term boost
## Transformation on dependent variable: RELATIVE RANKS
## ####################################################
##
## Call:
## lm(formula = as.formula(sprintf("perm_boost%s ~ %s", suffix,
##     predictors)), data = lmdata)
##
## Residuals:
##      Min       1Q   Median       3Q      Max
## -0.70715 -0.19327  0.02603  0.21907  0.59020
##
## Coefficients:
##                                  Estimate Std. Error t value Pr(>|t|)
## mean_before_relrank               0.116011   0.033760   3.436 0.000618 ***
## age_group70                       0.038062   0.022793   1.670 0.095301 .
## age_group20                       0.071226   0.094752   0.752 0.452437
## age_group30                       0.179281   0.084978   2.110 0.035174 *
```

```
## age_group40                        0.097324   0.048351   2.013 0.044444 *
## age_group50                       -0.009159   0.034750  -0.264 0.792173
## age_group60                       -0.008024   0.024690  -0.325 0.745276
## age_group80                        0.023841   0.022511   1.059 0.289860
## age_group90                        0.007153   0.030076   0.238 0.812081
## death_typeunnatural                0.122294   0.106773   1.145 0.252381
## genderFemale                       0.029356   0.025713   1.142 0.253903
## anglonon_anglo                    -0.034279   0.026503  -1.293 0.196225
## anglounknown                      -0.141044   0.030780  -4.582 5.29e-06 ***
## type_groupacademia/engineering     0.076314   0.070259   1.086 0.277705
## type_groupgeneral fame             0.003185   0.044332   0.072 0.942744
## type_groupknown for death         -0.024310   0.035778  -0.679 0.497031
## type_groupleadership              -0.101816   0.029326  -3.472 0.000543 ***
## type_groupsports                  -0.023928   0.029912  -0.800 0.423974
## age_group20:death_typeunnatural    0.090805   0.154766   0.587 0.557546
## age_group30:death_typeunnatural   -0.082225   0.151447  -0.543 0.587321
## age_group40:death_typeunnatural   -0.019321   0.134215  -0.144 0.885570
## age_group50:death_typeunnatural   -0.079483   0.127249  -0.625 0.532389
## age_group60:death_typeunnatural   -0.001916   0.125630  -0.015 0.987838
## age_group80:death_typeunnatural   -0.006991   0.193131  -0.036 0.971132
## age_group90:death_typeunnatural   -0.144877   0.297622  -0.487 0.626539
## ---
## Signif. codes:  0 '***' 0.001 '**' 0.01 '*' 0.05 '.' 0.1 ' ' 1
##
## Residual standard error: 0.2749 on 845 degrees of freedom
## Multiple R-squared:  0.1214, Adjusted R-squared:  0.0954
## F-statistic:  4.67 on 25 and 845 DF,  p-value: 8.867e-13
```

Models of news-vs.-Twitter boost difference for fixed person (without interactions)

```
##
##
## ########################################################
## REGRESSION MODEL
## Medium: NEWS vs. TWITTER
## Dependent variable: short-term boost
## Transformation on dependent variable: NONE
## ########################################################
##
## Call:
## lm(formula = as.formula(sprintf("peak_mean_boost%s_diff ~ %s",
##     suffix, predictors)), data = lmdata)
##
## Residuals:
##      Min       1Q   Median       3Q      Max
## -1.65313 -0.36479  0.00554  0.34318  2.14224
##
## Coefficients:
##                              Estimate Std. Error t value Pr(>|t|)
## (Intercept)                  -0.42705    0.04688  -9.109  < 2e-16 ***
## mean_before_relrank_diff     -0.21235    0.08346  -2.544  0.01112 *
## age_group20                  -0.57747    0.12582  -4.590 5.11e-06 ***
```

```
## age_group30                     -0.23483    0.12379  -1.897  0.05816 .
## age_group40                     -0.37445    0.09305  -4.024 6.22e-05 ***
## age_group50                     -0.20387    0.07324  -2.783  0.00550 **
## age_group60                     -0.17498    0.06057  -2.889  0.00396 **
## age_group80                      0.01435    0.05778   0.248  0.80388
## age_group90                      0.16419    0.07233   2.270  0.02346 *
## death_typeunnatural              0.34814    0.06995   4.977 7.82e-07 ***
## genderFemale                     0.09071    0.05323   1.704  0.08870 .
## anglonon_anglo                  -0.21941    0.05437  -4.036 5.93e-05 ***
## anglounknown                    -0.05238    0.06307  -0.831  0.40646
## type_groupacademia/engineering -0.04838    0.14619  -0.331  0.74076
## type_groupgeneral fame          -0.01644    0.09169  -0.179  0.85776
## type_groupknown for death        0.10452    0.07390   1.414  0.15760
## type_groupleadership             0.19975    0.06208   3.217  0.00134 **
## type_groupsports                 0.05875    0.06191   0.949  0.34292
## ---
## Signif. codes:  0 '***' 0.001 '**' 0.01 '*' 0.05 '.' 0.1 ' ' 1
##
## Residual standard error: 0.5712 on 852 degrees of freedom
## Multiple R-squared:  0.1013, Adjusted R-squared:  0.08336
## F-statistic: 5.648 on 17 and 852 DF,  p-value: 2.893e-12
##
##
##
## #####################################################
## REGRESSION MODEL
## Medium: NEWS vs. TWITTER
## Dependent variable: long-term boost
## Transformation on dependent variable: NONE
## #####################################################
##
## Call:
## lm(formula = as.formula(sprintf("perm_boost%s_diff ~ %s", suffix,
##     predictors)), data = lmdata)
##
## Residuals:
##      Min       1Q   Median       3Q      Max
## -1.25725 -0.05774  0.00741  0.06234  0.70460
##
## Coefficients:
##                                  Estimate Std. Error t value Pr(>|t|)
## (Intercept)                     -0.014983   0.014259  -1.051 0.293665
## mean_before_relrank_diff        -0.034104   0.025383  -1.344 0.179447
## age_group20                     -0.134769   0.038268  -3.522 0.000452 ***
## age_group30                     -0.089151   0.037649  -2.368 0.018110 *
## age_group40                     -0.101234   0.028300  -3.577 0.000367 ***
## age_group50                     -0.028959   0.022277  -1.300 0.193956
## age_group60                     -0.020521   0.018421  -1.114 0.265602
## age_group80                     -0.006351   0.017573  -0.361 0.717893
## age_group90                      0.015400   0.021998   0.700 0.484072
## death_typeunnatural             -0.008434   0.021275  -0.396 0.691877
## genderFemale                     0.028585   0.016189   1.766 0.077796 .
## anglonon_anglo                  -0.023095   0.016535  -1.397 0.162868
## anglounknown                     0.012408   0.019182   0.647 0.517899
```

```
## type_groupacademia/engineering -0.045961   0.044463  -1.034 0.301570
## type_groupgeneral fame          0.002200   0.027887   0.079 0.937150
## type_groupknown for death      -0.015268   0.022475  -0.679 0.497109
## type_groupleadership           -0.006173   0.018882  -0.327 0.743791
## type_groupsports                0.009053   0.018829   0.481 0.630790
## ---
## Signif. codes:  0 '***' 0.001 '**' 0.01 '*' 0.05 '.' 0.1 ' ' 1
##
## Residual standard error: 0.1737 on 852 degrees of freedom
## Multiple R-squared:  0.05249,    Adjusted R-squared:  0.03358
## F-statistic: 2.776 on 17 and 852 DF,  p-value: 0.0001535
##
##
##
## #####################################################
## REGRESSION MODEL
## Medium: NEWS vs. TWITTER
## Dependent variable: short-term boost
## Transformation on dependent variable: RELATIVE RANKS
## #####################################################
##
## Call:
## lm(formula = as.formula(sprintf("peak_mean_boost%s_diff ~ %s",
##     suffix, predictors)), data = lmdata)
##
## Residuals:
##     Min      1Q  Median      3Q     Max
## -0.6496 -0.1208  0.0038  0.1106  0.7404
##
## Coefficients:
##                                 Estimate Std. Error t value Pr(>|t|)
## (Intercept)                     0.007131   0.016294   0.438 0.661761
## mean_before_relrank_diff       -0.077831   0.029007  -2.683 0.007434 **
## age_group20                    -0.177328   0.043731  -4.055 5.47e-05 ***
## age_group30                    -0.091639   0.043024  -2.130 0.033461 *
## age_group40                    -0.112473   0.032340  -3.478 0.000531 ***
## age_group50                    -0.058774   0.025457  -2.309 0.021195 *
## age_group60                    -0.061181   0.021051  -2.906 0.003753 **
## age_group80                     0.012762   0.020081   0.636 0.525260
## age_group90                     0.072718   0.025139   2.893 0.003917 **
## death_typeunnatural             0.132598   0.024312   5.454 6.46e-08 ***
## genderFemale                    0.030131   0.018500   1.629 0.103744
## anglonon_anglo                 -0.077795   0.018896  -4.117 4.21e-05 ***
## anglounknown                   -0.017957   0.021921  -0.819 0.412906
## type_groupacademia/engineering -0.026667   0.050811  -0.525 0.599843
## type_groupgeneral fame         -0.011339   0.031869  -0.356 0.722071
## type_groupknown for death       0.028830   0.025684   1.123 0.261965
## type_groupleadership            0.060712   0.021578   2.814 0.005011 **
## type_groupsports                0.004572   0.021517   0.212 0.831794
## ---
## Signif. codes:  0 '***' 0.001 '**' 0.01 '*' 0.05 '.' 0.1 ' ' 1
##
## Residual standard error: 0.1985 on 852 degrees of freedom
## Multiple R-squared:  0.1042, Adjusted R-squared:  0.08629
```

```
## F-statistic: 5.827 on 17 and 852 DF,  p-value: 9.067e-13
##
##
##
## #####################################################
## REGRESSION MODEL
## Medium: NEWS vs. TWITTER
## Dependent variable: long-term boost
## Transformation on dependent variable: RELATIVE RANKS
## #####################################################
##
## Call:
## lm(formula = as.formula(sprintf("perm_boost%s_diff ~ %s", suffix,
##     predictors)), data = lmdata)
##
## Residuals:
##      Min       1Q   Median       3Q      Max
## -0.92186 -0.12959  0.01391  0.14260  0.95349
##
## Coefficients:
##                                 Estimate Std. Error t value Pr(>|t|)
## (Intercept)                     0.008043   0.022742   0.354  0.72367
## mean_before_relrank_diff       -0.230628   0.040485  -5.697 1.68e-08 ***
## age_group20                    -0.105519   0.061035  -1.729  0.08420 .
## age_group30                    -0.128012   0.060048  -2.132  0.03331 *
## age_group40                    -0.106048   0.045137  -2.349  0.01903 *
## age_group50                    -0.043230   0.035530  -1.217  0.22405
## age_group60                    -0.026262   0.029381  -0.894  0.37165
## age_group80                     0.004226   0.028027   0.151  0.88019
## age_group90                     0.080720   0.035086   2.301  0.02165 *
## death_typeunnatural             0.039642   0.033933   1.168  0.24303
## genderFemale                    0.007763   0.025820   0.301  0.76373
## anglonon_anglo                 -0.074869   0.026373  -2.839  0.00464 **
## anglounknown                    0.023978   0.030594   0.784  0.43341
## type_groupacademia/engineering -0.069726   0.070916  -0.983  0.32578
## type_groupgeneral fame          0.003712   0.044479   0.083  0.93352
## type_groupknown for death       0.001229   0.035846   0.034  0.97266
## type_groupleadership            0.066667   0.030116   2.214  0.02711 *
## type_groupsports                0.008463   0.030031   0.282  0.77816
## ---
## Signif. codes:  0 '***' 0.001 '**' 0.01 '*' 0.05 '.' 0.1 ' ' 1
##
## Residual standard error: 0.2771 on 852 degrees of freedom
## Multiple R-squared:  0.0743, Adjusted R-squared:  0.05583
## F-statistic: 4.023 on 17 and 852 DF,  p-value: 9.057e-08
```

Models of news-vs.-Twitter boost difference for fixed person (with "age by manner of death" interaction)

```
##
##
## #####################################################
## REGRESSION MODEL
```

```
## Medium: NEWS vs. TWITTER
## Dependent variable: short-term boost
## Transformation on dependent variable: NONE
## #####################################################
##
## Call:
## lm(formula = as.formula(sprintf("peak_mean_boost%s_diff ~ %s",
##     suffix, predictors)), data = lmdata)
##
## Residuals:
##      Min       1Q   Median       3Q      Max
## -1.65119 -0.36422  0.00888  0.34055  2.14507
##
## Coefficients:
##                                 Estimate Std. Error t value Pr(>|t|)
## mean_before_relrank_diff        -0.21682    0.08400  -2.581  0.01001 *
## age_group70                     -0.42927    0.04758  -9.022  < 2e-16 ***
## age_group20                     -0.82078    0.19722  -4.162 3.48e-05 ***
## age_group30                     -0.80494    0.17718  -4.543 6.35e-06 ***
## age_group40                     -0.78266    0.10074  -7.769 2.28e-14 ***
## age_group50                     -0.63262    0.07226  -8.755  < 2e-16 ***
## age_group60                     -0.60094    0.05132 -11.711  < 2e-16 ***
## age_group80                     -0.41640    0.04659  -8.938  < 2e-16 ***
## age_group90                     -0.26085    0.06271  -4.160 3.51e-05 ***
## death_typeunnatural              0.40697    0.22256   1.829  0.06782 .
## genderFemale                     0.09575    0.05356   1.788  0.07419 .
## anglonon_anglo                  -0.21719    0.05509  -3.942 8.74e-05 ***
## anglounknown                    -0.05533    0.06348  -0.872  0.38367
## type_groupacademia/engineering  -0.04801    0.14652  -0.328  0.74326
## type_groupgeneral fame          -0.01437    0.09234  -0.156  0.87634
## type_groupknown for death        0.10249    0.07432   1.379  0.16825
## type_groupleadership             0.19868    0.06232   3.188  0.00148 **
## type_groupsports                 0.05601    0.06229   0.899  0.36880
## age_group20:death_typeunnatural -0.32913    0.32231  -1.021  0.30746
## age_group30:death_typeunnatural  0.17762    0.31605   0.562  0.57426
## age_group40:death_typeunnatural -0.11676    0.28006  -0.417  0.67686
## age_group50:death_typeunnatural -0.04974    0.26513  -0.188  0.85125
## age_group60:death_typeunnatural -0.06810    0.26168  -0.260  0.79474
## age_group80:death_typeunnatural  0.16940    0.40237   0.421  0.67385
## age_group90:death_typeunnatural -0.37706    0.61904  -0.609  0.54262
## ---
## Signif. codes:  0 '***' 0.001 '**' 0.01 '*' 0.05 '.' 0.1 ' ' 1
##
## Residual standard error: 0.5724 on 845 degrees of freedom
## Multiple R-squared:  0.4346, Adjusted R-squared:  0.4178
## F-statistic: 25.98 on 25 and 845 DF,  p-value: < 2.2e-16
##
##
##
## #####################################################
## REGRESSION MODEL
## Medium: NEWS vs. TWITTER
## Dependent variable: long-term boost
## Transformation on dependent variable: NONE
```

```
## ######################################################
##
## Call:
## lm(formula = as.formula(sprintf("perm_boost%s_diff ~ %s", suffix,
##     predictors)), data = lmdata)
##
## Residuals:
##      Min       1Q   Median       3Q      Max
## -1.20604 -0.05399  0.00862  0.05958  0.65987
##
## Coefficients:
##                                  Estimate Std. Error t value Pr(>|t|)
## mean_before_relrank_diff        -0.0387605  0.0254315  -1.524  0.12785
## age_group70                     -0.0127546  0.0144053  -0.885  0.37618
## age_group20                     -0.0266540  0.0597136  -0.446  0.65545
## age_group30                     -0.1643295  0.0536460  -3.063  0.00226 **
## age_group40                     -0.1367607  0.0305002  -4.484 8.34e-06 ***
## age_group50                     -0.0394232  0.0218780  -1.802  0.07191 .
## age_group60                     -0.0364275  0.0155368  -2.345  0.01928 *
## age_group80                     -0.0201342  0.0141055  -1.427  0.15383
## age_group90                      0.0010249  0.0189868   0.054  0.95697
## death_typeunnatural             -0.0496287  0.0673856  -0.736  0.46164
## genderFemale                     0.0285184  0.0162164   1.759  0.07900 .
## anglonon_anglo                  -0.0235607  0.0166806  -1.412  0.15818
## anglounknown                     0.0105550  0.0192186   0.549  0.58301
## type_groupacademia/engineering  -0.0463112  0.0443633  -1.044  0.29683
## type_groupgeneral fame           0.0001484  0.0279576   0.005  0.99577
## type_groupknown for death       -0.0162957  0.0225018  -0.724  0.46915
## type_groupleadership            -0.0049896  0.0188673  -0.264  0.79149
## type_groupsports                 0.0050388  0.0188582   0.267  0.78939
## age_group20:death_typeunnatural -0.1369124  0.0975852  -1.403  0.16098
## age_group30:death_typeunnatural  0.1441439  0.0956893   1.506  0.13234
## age_group40:death_typeunnatural  0.1064914  0.0847944   1.256  0.20951
## age_group50:death_typeunnatural  0.0275709  0.0802742   0.343  0.73134
## age_group60:death_typeunnatural  0.0599112  0.0792287   0.756  0.44975
## age_group80:death_typeunnatural  0.0063933  0.1218248   0.052  0.95816
## age_group90:death_typeunnatural  0.0029407  0.1874274   0.016  0.98749
## ---
## Signif. codes:  0 '***' 0.001 '**' 0.01 '*' 0.05 '.' 0.1 ' ' 1
##
## Residual standard error: 0.1733 on 845 degrees of freedom
## Multiple R-squared:  0.101,  Adjusted R-squared:  0.07445
## F-statistic: 3.799 on 25 and 845 DF,  p-value: 1.803e-09

##
##
## ######################################################
## REGRESSION MODEL
## Medium: NEWS vs. TWITTER
## Dependent variable: short-term boost
## Transformation on dependent variable: RELATIVE RANKS
## ######################################################
##
## Call:
```

```
## lm(formula = as.formula(sprintf("peak_mean_boost%s_diff ~ %s",
##     suffix, predictors)), data = lmdata)
##
## Residuals:
##      Min       1Q   Median       3Q      Max
## -0.64846 -0.11978  0.00327  0.10887  0.74189
##
## Coefficients:
##                                 Estimate Std. Error t value Pr(>|t|)
## mean_before_relrank_diff        -0.079660   0.029192  -2.729 0.006487 **
## age_group70                      0.005902   0.016535   0.357 0.721241
## age_group20                     -0.131089   0.068543  -1.913 0.056148 .
## age_group30                     -0.142074   0.061578  -2.307 0.021283 *
## age_group40                     -0.100671   0.035010  -2.876 0.004135 **
## age_group50                     -0.052507   0.025113  -2.091 0.036838 *
## age_group60                     -0.051830   0.017834  -2.906 0.003754 **
## age_group80                      0.018036   0.016191   1.114 0.265611
## age_group90                      0.080562   0.021794   3.696 0.000233 ***
## death_typeunnatural              0.155420   0.077349   2.009 0.044819 *
## genderFemale                     0.032534   0.018614   1.748 0.080860 .
## anglonon_anglo                  -0.076134   0.019147  -3.976  7.6e-05 ***
## anglounknown                    -0.018445   0.022060  -0.836 0.403316
## type_groupacademia/engineering  -0.026851   0.050923  -0.527 0.598128
## type_groupgeneral fame          -0.010738   0.032091  -0.335 0.738007
## type_groupknown for death        0.027646   0.025829   1.070 0.284766
## type_groupleadership             0.060264   0.021657   2.783 0.005512 **
## type_groupsports                 0.004710   0.021646   0.218 0.827786
## age_group20:death_typeunnatural -0.081471   0.112014  -0.727 0.467224
## age_group30:death_typeunnatural  0.071281   0.109837   0.649 0.516537
## age_group40:death_typeunnatural -0.038913   0.097332  -0.400 0.689404
## age_group50:death_typeunnatural -0.020927   0.092143  -0.227 0.820392
## age_group60:death_typeunnatural -0.046500   0.090943  -0.511 0.609270
## age_group80:death_typeunnatural  0.071107   0.139837   0.508 0.611238
## age_group90:death_typeunnatural -0.166528   0.215139  -0.774 0.439120
## ---
## Signif. codes:  0 '***' 0.001 '**' 0.01 '*' 0.05 '.' 0.1 ' ' 1
##
## Residual standard error: 0.1989 on 845 degrees of freedom
## Multiple R-squared:  0.1079, Adjusted R-squared:  0.08154
## F-statistic: 4.089 on 25 and 845 DF,  p-value: 1.466e-10
##
##
##
## #####################################################
## REGRESSION MODEL
## Medium: NEWS vs. TWITTER
## Dependent variable: long-term boost
## Transformation on dependent variable: RELATIVE RANKS
## #####################################################
##
## Call:
## lm(formula = as.formula(sprintf("perm_boost%s_diff ~ %s", suffix,
##     predictors)), data = lmdata)
##
```

```
## Residuals:
##      Min       1Q   Median       3Q      Max
## -0.92420 -0.13055  0.01538  0.14045  0.95719
##
## Coefficients:
##                                   Estimate Std. Error t value Pr(>|t|)
## mean_before_relrank_diff        -0.2341465  0.0407524  -5.746 1.28e-08 ***
## age_group70                      0.0106514  0.0230835   0.461  0.64461
## age_group20                      0.0031530  0.0956873   0.033  0.97372
## age_group30                     -0.1718587  0.0859643  -1.999  0.04591 *
## age_group40                     -0.1054249  0.0488746  -2.157  0.03128 *
## age_group50                     -0.0333427  0.0350581  -0.951  0.34184
## age_group60                     -0.0213921  0.0248968  -0.859  0.39046
## age_group80                      0.0133539  0.0226032   0.591  0.55481
## age_group90                      0.0899990  0.0304251   2.958  0.00318 **
## death_typeunnatural             -0.0135298  0.1079812  -0.125  0.90032
## genderFemale                     0.0080192  0.0259857   0.309  0.75770
## anglonon_anglo                  -0.0751648  0.0267296  -2.812  0.00504 **
## anglounknown                     0.0227608  0.0307967   0.739  0.46007
## type_groupacademia/engineering  -0.0697905  0.0710894  -0.982  0.32651
## type_groupgeneral fame           0.0031289  0.0448002   0.070  0.94434
## type_groupknown for death       -0.0002293  0.0360578  -0.006  0.99493
## type_groupleadership             0.0668489  0.0302337   2.211  0.02730 *
## type_groupsports                 0.0050713  0.0302191   0.168  0.86677
## age_group20:death_typeunnatural -0.0923192  0.1563740  -0.590  0.55510
## age_group30:death_typeunnatural  0.1416421  0.1533360   0.924  0.35589
## age_group40:death_typeunnatural  0.0782650  0.1358777   0.576  0.56477
## age_group50:death_typeunnatural  0.0504094  0.1286343   0.392  0.69524
## age_group60:death_typeunnatural  0.0908410  0.1269589   0.716  0.47449
## age_group80:death_typeunnatural  0.0187045  0.1952165   0.096  0.92369
## age_group90:death_typeunnatural -0.0496572  0.3003405  -0.165  0.86872
## ---
## Signif. codes:  0 '***' 0.001 '**' 0.01 '*' 0.05 '.' 0.1 ' ' 1
##
## Residual standard error: 0.2777 on 845 degrees of freedom
## Multiple R-squared:  0.07775,    Adjusted R-squared:  0.05046
## F-statistic: 2.849 on 25 and 845 DF,  p-value: 4.86e-06
```

Comprehensiveness of Spinn3r

Although Spinn3r does not publicly disclose its data collection strategy, we confirm the comprehensiveness of the corpus as follows. The news portion of the Spinn3r corpus contains documents from each of the 6,608 English-language news domains identified externally via Google News, which hints at Spinn3r's completeness with respect to the entirety of online news. Moreover, in each of the four years (2010–2013) that are covered in their entirety by our study period, the average daily number of English tweets in the Spinn3r corpus amounts to 8–19% of Twitter's full tweet volume across all languages.[1] With English tweets estimated to have accounted for 53% of all tweets in 2010[2] and 34% in 2013[3], we estimate that the Spinn3r corpus contains about one third of all English tweets, as summarized in the following table:

Year	Full Twitter All tweets[1]	Spinn3r English tweets	Full Twitter English tweets[2,3]	Completeness English tweets
2010	35 million/day	6.7 million/day	19 million/day	36%
2011	200 million/day	20 million/day		
2012	340 million/day	28 million/day		
2013	500 million/day	51 million/day	170 million/day	30%

Supplementary Table 3: Completeness estimation of the English-language portion of the Spinn3r corpus, with respect to the entirety of English-language tweets on full Twitter.

[1] https://www.internetlivestats.com/twitter-statistics/

[2] B. Poblete, R. Garcia, M. Mendoza, A. Jaimes. Do all birds tweet the same? Characterizing Twitter around the world. In *Proceedings of the 20th ACM International Conference on Information and Knowledge Management.* pp. 1025–1030 (2011).

[3] http://www.statista.com/statistics/267129/most-used-languages-on-twitter/
